# Exploratory Data Mining for Subgroup Cohort Discoveries and Prioritization

**DOI:** 10.1109/JBHI.2019.2939149

**Published:** 2019-09-05

**Authors:** Danlu Liu, William Baskett, David Beversdorf, Chi-Ren Shyu

**Affiliations:** Department of Electrical Engineering and Computer Science, University of Missouri, Columbia, MO 65211 USA; Institute for Data Science and Informatics, University of Missouri, Columbia, MO 65211 USA; Departments of Radiology, Neurology, and Psychological Sciences, and with the Thompson Center for Autism and Neurodevelopmental Disorders, University of Missouri, Columbia, MO 65211 USA; Institute for Data Science and Informatics, Department of Electrical Engineering and Computer Science, and with the School of Medicine, University of Missouri, Columbia, MO 65211 USA

**Keywords:** Contrast mining, exploratory mining, patient cohort identification, subgroup discovery

## Abstract

Finding small homogeneous subgroup cohorts in large heterogeneous populations is a critical process for hypothesis development in biomedical research. Concurrent computational approaches are still lacking in robust answers to the question “what hypotheses are likely to be novel and to produce clinically relevant results with well thought-out study designs?” We have developed a novel subgroup discovery method which employs a deep exploratory mining process to slice and dice thousands of potential subpopulations and prioritize potential cohorts based on their explainable contrast patterns and which may provide interventionable insights. We conducted computational experiments on both synthesized data and a clinical autism data set to assess performance quantitatively for coverage of pre-defined cohorts and qualitatively for novel knowledge discovery, respectively. We also conducted a scaling analysis using a distributed computing environment to suggest computational resource needs for when the subpopulation number increases. This work will provide a robust data-driven framework to automatically tailor potential interventions for precision health.

## I. Introduction

MUCH of successful biomedical research relies on identifying key predictive factors within specific populations [1]. Discovering subgroups within a large-scale population and being able to explain what differentiates them from that population is essential in precision medicine or designing relevant clinical trials. The National Academy of Medicine [2] urges the research community to target high-need patients from smaller homogeneous subgroups for precision health with better outcomes. Moreover, today, studies of randomized clinical trials and meta-analysis of literature suggest that six of the top ten highest-grossing drugs in the US are effective for less than 12% of patients and even the most effective drugs from that list have positive outcomes in only 25% of patients [1]. This “imprecision medicine” practice not only harms certain populations of patients, it also burdens the healthcare system financially. While there are complex issues related to the ineffectiveness of these drugs, using data analytics methods will streamline the drug development process by guiding it with data-driven evidence [3]. By finding meaningful and homogeneous subgroups prior to conducting clinical trials, researchers can further study focused populations and identify potential risk factors from complex data sources to create tailored treatments [4]. In fact, it is rare for a clinical trial hypothesis to be “spot-on” for a large group of patients due to complex combinations of ethnic, demographic, genetic, chronic, behavioral, and environmental specificities. Many medical discoveries have been byproducts of failed clinical trials that, while producing disappointing overall results, revealed surprising responsiveness from certain patient subgroups during post-trial analyses [1]. However, there are two major barriers to such tailored care: the effort required to identify meaningful subgroups of patients for clinical trials/outcome research, and the high cost of developing interventions for such small populations. These barriers go hand in hand due to the complexity and resources needed to efficiently identify subgroups and the possibility to repurpose interventions, such as drug repositioning.

In addition to manually defining cohorts, many techniques have been developed to identify cohorts from a large population [5]. The premise of the existing cohort discovery methods often starts with a pre-defined pair of populations, such as diseased and non-diseased groups, and then discovers cohorts from the populations. Machine learning approaches could be potential solutions for tackling this subgroup cohort discovery task [6]. One branch of the methods is rule-based cohort discovery. Lee *et al*. [7] used discriminant analysis and Niemann *et al*. [8] applied SD-Map algorithm [9] and hierarchical clustering to automatically create rules to classify the pre-defined populations. The second branch of methods applied machine learning algorithms for disease prediction or risk factors discovery. Hielscher *et al*. [10] introduced a constraint-based subspace clustering algorithm called DRESS to discover and score candidate spaces on an epidemiological cohort study. Li *et al*. [11] used topology-based networks to cluster subtypes of type 2 diabetes. Although the latest developments in deep learning approaches have been extensively and successfully applied in speech recognition [12], computer vision [13], radiology [14], and many additional health-related applications in recent years [15], those black box models are valuable in applications where reasoning is not necessary. However, in biomedical research and health care applications, high-level explanations are critical and the recent enhancements in deep learning to improve interpretability, such as attention mechanism [16], influence functions [17], are still insufficient to allow for potential intervention.

**Fig. 1.**
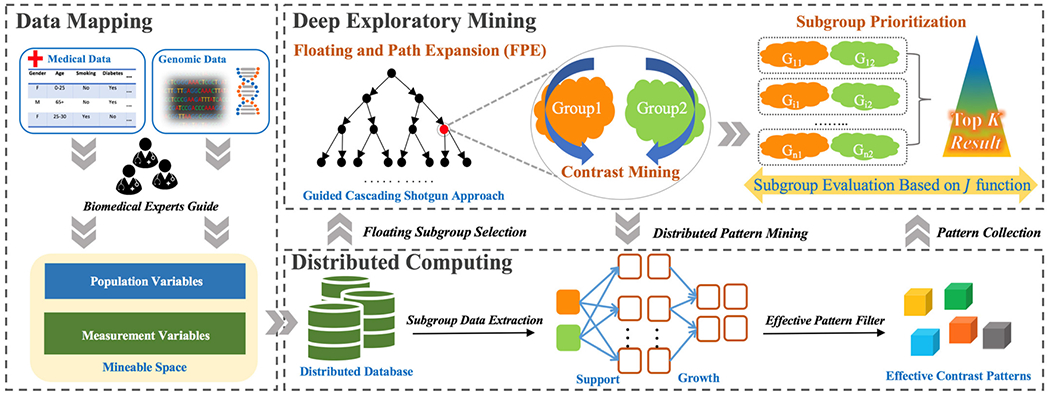
The overall system architecture of the distributed exploratory mining workflow. The architecture can be divided into three parts—data mapping, deep exploratory mining and distributed computing. The expert is involved in the data mapping part to map raw data into the mineable space, then the formatted data is fed to the deep exploratory mining process using a Big Data ecosystem. Contrast subgroups are selected and their contrast patterns are mined in the distributed environment. Finally, all selected contrast subgroups are evaluated based on their effective contrast patterns using an evaluation function *J*.

To bridge the knowledge gap, in this paper, we introduce a unique exploratory mining approach, shown in Fig. 1, that enables the broad biomedical research community to answer the following questions: *Which subgroups of patients might benefit from interventions that are likely to be effective for the selected populations*? Our contribution is the development of a suite of computational methods that are pipelined in a distributed computing environment to tackle the issues of identifying and prioritizing cohorts of patient subpopulations and revealing explainable contrast patterns for potential interventions. The impact of this work is to allow researchers and clinicians to intelligently slice and dice through hundreds of thousands of potential subgroups and focus on only those subgroups which are evidence-based, data-driven, and statistically significant with actionable potential. We believe this capability will enable the biomedical research community to acquire advanced medical knowledge and produce innovative treatments at a much faster pace than what is currently possible.

## II. Related Works

In the field of data mining, prominent contributions have been made by researchers in three categories of methods, namely subgroup discovery [18], contrast mining [19] and contrast set mining [20] to identify significant subgroups or reveal the differences between two or more subgroups using supervised rule learning [21]. The first category of data mining methods in subgroup discovery aims to identify subgroups [22] in the form of *Cond* ⇒ *Target_value_* [23] for a predefined user-specific population (*Target_value_*). This category of subgroup discovery attempts to explore the combinatory space to detect a meaningful cause that leads to the target population or a general description of that target population (*Cond*). Frequent pattern mining-based methods, such as Apriori-SD [24] and SD-Map [9] have been applied in the subgroup discovery process to prune the exploratory space and reduce computational complexity. The second category of methods in contrast mining attempts to identify contrast patterns (CPs) of features which differentiate two groups by exploring patterns which have an imbalanced prevalence between the groups [25]. The initial method to discover contrast patterns was proposed by Dong *et al*. using the property of borders to mine frequent contrast patterns and the concept of ‘jumping emerging patterns’ for classifications [25]. Techniques using emerging patterns and jumping emerging patterns have been utilized in many areas, such as bioinformatics [26] and chemical modeling [27]. Contrast patterns can also be extracted using tree structures to shorten the computation time, such as ratio tree [28] and CP-tree [29]. The third category of methods in contrast set mining attempts to discover the differences between several subgroups [20]. It requires the user to specify a list of subgroups *G*_1_, *G*_2_, … , *G_n_* to extract the combinations of characteristics that differentiate the groups from each other [30]. Contrast set mining was first introduced by Stephen *et al*. who reported a framework called STUCCO [20] which allowed for the exploration of the contrast set space using a breadth-first strategy and heuristic pruning rules to reduce the search space to a manageable size. However, both clusters of methods in contrast mining and contrast set mining are limited to finding the differences between pre-defined subgroups.

In addition, traditional statistical analysis, such as logistic regression model, has been widely used [5]. Furthermore, with the utilization of natural language processing (NLP) tool to extract terms or concept from clinical text, the accuracy of cohort retrieval and identification increases significantly compared to using structured data alone [31].

Conversely, unsupervised clustering [32] and network analysis methods [11] add the capability of discovering sub-clusters from the data without preset class labels. However, while they are valuable in many biomedical applications, there are still limitations for two reasons: (1) sub-clusters are discovered based on degree of separation without taking into consideration the characteristics of each cluster to form control and treatment groups; and (2) there are combinatorial explosion issues involved with identifying clusters of subgroups from all potential subpopulations, which can result in hundreds of thousands or even millions of all potential groups.

From what is available in the computing community, subgroup discovery, contrast mining and contrast set mining all require a clear and pre-defined target. This limits the impact of discovery results particularly in biomedicine where the successful assessment of explainable interventions from viable subgroups plays a key role in advancing the field. In this paper, our definition of cohort is broader than the setting used in the traditional cohort discovery research since our work is to discover new target populations which are normally pre-defined in the previous approaches. In the remainder of this paper, we will interchangeably use cohort and population subgroup for the discussions of the algorithm and computational experiments.

The rest of this paper is organized as follows. Section III introduces mapping raw biomedical data into clinically explainable and mineable space. Section IV describes the algorithm for the deep exploratory mining process consisting of the Floating and Path Expansion approach, effective contrast pattern extraction, and subgroup prioritization using *J*-value. Section V illustrates a distributed computing algorithm, which is necessary due to the large search space in subgroup selection and pattern extraction, to streamline the mining process, Section VI reports results of computational experiments on data sets from synthesized test sets and biomedical datasets in autism spectrum disorder. Section VII concludes the results and discusses future work.

## III. Data Mapping

To ensure the meaningfulness of data analytics results, we utilize the population, intervention, comparison, and outcome (PICO) guideline [33] to map raw data into mineable information that resembles the key components of the biomedical research hypothesis generation procedure which should be targeting a concrete research direction with a high-level hypothesis. As shown in Fig. 1, raw data can be extracted from electronic health records, biomedical images, or genomics data during the data mapping process. This process defines two types of variables: population variables (*P*) to divide patient populations into subgroups and measurement variables (*M*) to describe the main characteristics (patterns) of the subgroups. Depending on the research question, the population variable can include co-morbidities and chronic conditions while measurement variables may include lab results, intervention procedures, device signals, single nucleotide polymorphisms (SNPs), expression data, etc. In this work, we assume all the variables in the dataset contain only categorical attributes. For all types of variables, the categorization of variables is based on the literature or known grouping guidelines, such as age, BMI, glucose level, etc. For variables lacking categorization guidelines that are clinically meaningful, we select methods that are appropriate for the domain, such as equal-width, equal-density, entropy-based, or adjacent pairs-based algorithms. To deal with missing data, we performed multiple pre-processing steps: 1. For the genomic dataset, genotype imputation is a commonly used method in gene association studies [34]. We used Beagle (version 4.1), a bioinformatics tool, to infer genotypes that were missing from our data [35], [36]. 2. For phenotype fields, we omitted patients with too many missing values and used ‘NA’ as a new category to represent missing values if that variable does not have too many missing values. However, ‘NA’ variables are never used to form subgroups.

Given a collection of population variables *P* = {*P*_1_, *P_2_*, … , *P_n_*}, each variable *P_i_* has a set of categories *C_i_* = {*C*_*i*,1_, *C*_*i*,2_, … , *C_i,i_k__*}, where 1 ≤ *i* ≤ *n* and *i_k_* is the cardinality of *C_i_*. For each population variable *P_i_*, any two exclusive category values *C_i,m_*, *C_i,n_* ∈ *C_i_* can form a contrast subgroup pair (*C_i,m_* ↔ *C_i,n_*). By adding a new inclusion constraint *P_j_* with its pair (*C_j,k_* ↔ *C_j,l_*), *C_j,k_*, *C_j,l_* ∈ *C_j_*, to the original subgroup selection criterion, a more focused subgroup pair ((*C_i,m_* Λ *C_j,k_*) ↔ (*C_i,n_* Λ *C_j,l_*)) can be formed. The contrast subgroup can be described using a contrast-subgroup pair in the form of (*C_i,m_* Λ … Λ *C_j,k_*) ↔ (*C_i,n_* Λ … Λ *C_j,l_*), where *C_i,m_*, *C_i,n_* and *C_j,k_*, *C_j,l_* are categories from population variables *P_i_* and *P_j_*, respectively. The first term of a contrast subgroup (*C_i,m_* Λ … Λ *C_j,k_*) describes the traits of the first group and the second term describes the traits of the second group. For example, (*Female* Λ *Young*) ↔ (*Male* Λ *Young*) is a contrast-subgroup pair comparing young females versus young males. It is described by two population variables in gender and age. *Female*, *Male* are categories of gender and *Young* is a category of age. The contrast subgroup must satisfy two conditions:

- The categories in the first and second subgroups are in one-to-one positional correspondence. (i.e., the *i*th values in the first and second subgroup are both the category values of the population variable *i*.)
- The population variables in each subgroup are exclusively distinct. (i.e., the population variable can be used at most once within each subgroup.)

Condition (a) guarantees that the two groups are comparable in clinical applications to target cohorts of patients that meet the recruitment criteria for clinical trials. Condition (b) ensures selected subgroups have exclusive patient samples. In many biomedical research questions, subgroup pairs often share common population categories except only one or a limited number of categories that make studies manageable and controllable, for example ((*Female* Λ *Young*) ↔ (*Male* Λ *Young*)) with the shared population category *Young* of age population variable. This data-mapping pipeline is developed to take a raw data file and a data definition file (assigning types and potential categories for each variable) to create a mineable data source for the deep exploratory mining process. This pipeline is designed to be generic to handle multiple genotype and phenotype data formats.

## IV. Deep Exploratory Mining

In this section, we introduce the methods underpinning the Deep Exploratory Mining Process to automatically crawl a large number of subgroups from the entire population space and to result in a sizable candidate pool of patient population subgroups. As shown in Fig. 1, the process consists of the following three components.

### A. Floating and Path Expansion

This module provides a three-level algorithmic approach. The top-level method, *Guided Cascading Shotgun*, applies a large number of second-level *Floating Contrast Subgroup Selection* processes, each of which is supported by a series of third-level *Inclusion* and *Exclusion* procedures.

Given *n_p_* population variables with an average of *n_c_* categories per variable (e.g., blood pressure (BP) variable has *n_BP_* = 4 categories based on the American College of Cardiology (ACC) guideline [37]), there are $n_{c}^{n_{p}}$ potential subgroups. This number could grow to an unmanageable scale. Therefore, the core of the subgroup selection process is to efficiently and automatically identify candidate pairs of subgroups to target certain patient subgroups. The algorithm executes an extended floating selection process [38], which executes a series of inclusion and exclusion processes and is expected to provide solutions closer to the global optima than a “greedy” approach can achieve [39], based on the assessment of the quality of contrast patterns between pairs of subgroups. This extended approach features a unique pair of inclusion and exclusion functions that are designed to assess contrasts between cohorts.

As shown in Algorithm 1, Lines 2-6 call the INCLUSION function to choose a base for the floating selection as an initiation step. Then the algorithm alternatively executes a series of inclusion (Lines 8-9), exclusion (Lines 10-15), and continue exclusion processes (Lines 16-22) which are based on assessments of the quality and quantity of contrast patterns between a pair of contrast subgroups for the selected population variables. As shown in the INCLUSION function, Lines 3-4 of the function use categories *C_i_* = {*C*_*i*,1_, *C*_*i*,2_, … , *C_i,n_*} of a population variable *P_i_* ∈ *P*(*D*) to generate the contrast subgroup pair set *CPair_i_* = {(*C*_*i*,1_ ↔ *C*_*i*,2_), (*C*_*i*,1_ ↔ *C*_*i*,3_) … , (*C*_*i,n*−1_ ↔ *C_i,n_*)}. Lines 5-6 of the INCLUSION function describe how each pair (*C_i,m_*, *C_i,n_*) is added to the selected contrast subgroup to form a temporary selected contrast subgroup (*SCG_temp_*). Incidentally, the selected contrast group *SCG_temp_* is in the form of (*C_i,m_* Λ … Λ *C_j,k_*) ↔ (*C_i,n_* Λ … Λ *C_j,l_*), where *C_i,m_*, *C_i,n_* and *C_j,k_*, *C_j,l_* are categories from population variables *P_i_* and *P_j_*, respectively. The entire population *S* is split into three subgroups based on *SCG*_*temp*_ (Line 7 of the INCLUSION function)–a pair of contrasting subgroups *S*_*G*1_, *S*_*G*2_ and the outer group of remaining populations *S*_*outer*_ = *S* − {*S*_*G*1_, *S*_*G*2_}. In the next two sections, we will focus on methods of extracting contrast patterns from the pair of subgroups *S*_*G*1_, *S*_*G*2_. To ensure the patterns are truly unique in the selected subgroups, their prevalence within the subgroups has to be statistically significant in comparison with the outer group. To evaluate the significant difference between the pair of subgroups, an assessment function calculates the *J* value (to be formulated in the next subsection) after contrast patterns are mined (Lines 8-9 of the INCLUSION function). Lines 12-13 of the function choose the selected contrast group with the highest *J* value (*SCG*_*highest*_) as the best contrast subgroup and update *SCG* with it. If the selected population variable is added to the population variable list, it will not be considered in the later inclusion process (Line 14). Similarly, the EXCLUSION function loops over the selected contrast subgroup *SCG* and excludes each contrast subgroup pair to form a temporary selected contrast group *SCG*_*temp*_ and calculate its *J* value as shown in Lines 3-8 of the EXCLUSION function. If removing a population variable results in the highest *J* gain, as shown in Line 11, the EXCLUSION function will drop the variable from the subgroup inclusion list. This iterative process performs the inclusion and exclusion steps alternately with a stop criterion (*J*(*k*) − *J*(*k* − 1))/*J*(*k*) ≤ *α* for iteration *k* or the number of contrast subgroup variables is greater than maximal number of variables *l* as shown at Line 7 in Algorithm 1.

**Fig. 2.**
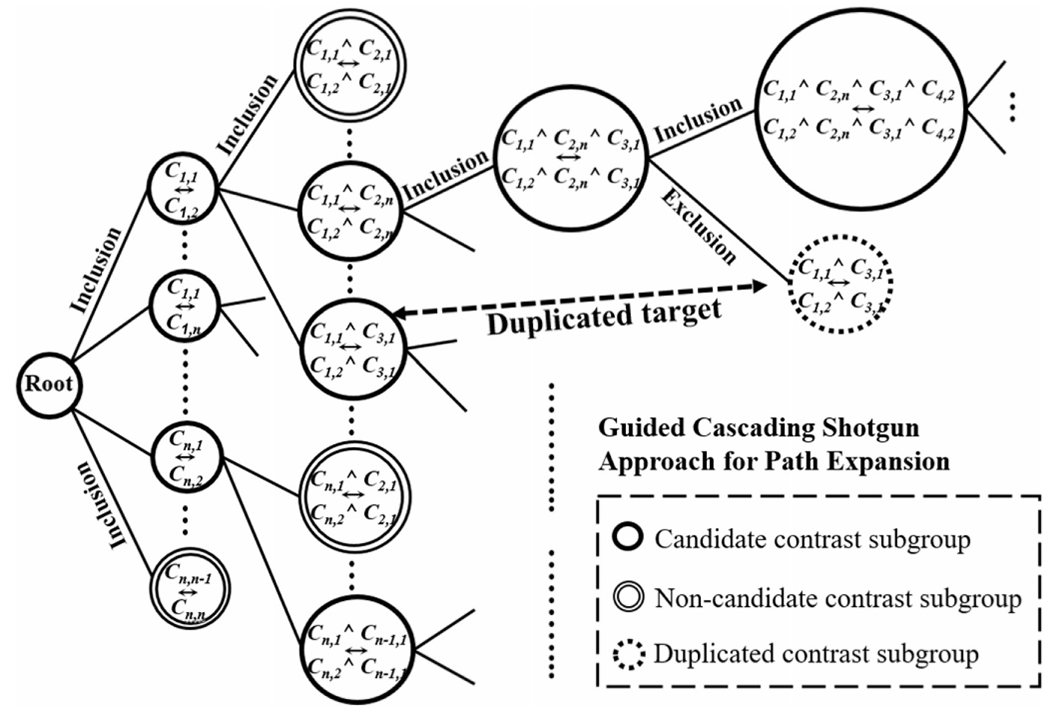
The Guided Cascading Shotgun approach for the path expansion process, which explores multiple paths in each inclusion and exclusion procedure for cohort selection.

The traditional floating selection algorithm [38] picks the best variable resulting in the highest evaluation value (*J*_*highest*_) to include or exclude a population variable for the improvement of the objective goal through an iterative process to find an optimal solution, which is likely to be local. However, in biomedical discoveries, identifying a single cohort of patients for clinical trials is neither sufficient nor realistic. Taking advantage of the advancement of computing power, we have developed the *Guided Cascading Shotgun* (GCS) approach to explore hundreds to thousands of potential subgroup cohorts which are comparably valuable during the *Floating Contrast Subgroup Selection* process. This GCS approach, which features deep exploration of the search space, is different from the traditional floating selection process, which seeks for a single suboptimal solution. As shown in Fig. 2, the *Path Expansion* process will explore multiple paths. Starting from a root node with an empty *SCG*, the algorithm forms several contrast subgroup pairs (*C_i,m_* ↔ *C_i,n_*) based on any two exclusive category values *C_i,m_*, *C_i,n_* for population variable *P_i_*. This approach then allows the subgroup discovery process to explore many potential paths (pellets in a shell) using an expanding factor *p* ∈ [0, 1]. The total number of candidate paths is determined by the following:

(1) $$N_{\mathrm{track}}=Max\left\{\left|S_{{\mathrm{more}}_{J}}\right|,\left|S_{{high}_{J}}\right|\right\},$$

where |*S*_*more_J_*_| = ⌈*n* * *p*⌉ is the number of paths for top (100 * *p*)% from all *n* possible paths and *S_high_J__* = {*n_i_*| *J*(*n_i_*) ≥ *J_highest_* * (1 − *p*), *i* = 1, 2 , … , *n*} is the number of paths where *J* values are among the top (100 * *p*)% of the highest *J* value for all *n* possible paths. *N*_*track*_ takes into consideration both the quantity and quality of the candidate paths. Each candidate path (pellet) in the second layer is represented by a solid circle and will then continue the exploration process through another layer of inclusion and exclusion processes to add (the pellet upgrades to a shell and then aims at the next layer of targets) or remove one population variable. A population variable selection tree is built to track which variables and categories are selected for subgroup comparisons. While the non-candidate paths indicated by double solid circles will not be expanded anymore.

**Algorithm 1:** Floating Contrast Subgroup Selection.

**Inputs:**	
*P*(*D*): Population variable set for dataset *D*.	
*J*(*k*): Contrast between subgroups with *k* variables.	
*α*: stopping criteria for the algorithm.	
*l*: maximal number of population variables for contrast subgroups.	
**Output:** *Selected Contrast Group SCG*	
1:	*SCG* ← *ϕ*; *k* ← 0; *J*(*k*) ← 0;
2:	// **Initiation Step:**
3:	**WHILE** *k* < 2 **DO**
4:	INCLUSION (*P*(*D*), *SCG*)
5:	*k* ← *k* + 1
6:	**END**
7:	**WHILE** ((*J*(*k*) − *J*(*k* − 1))/*J*(*k*) > *α* AND *k* < *l* **DO**
8:	// **Inclusion Procedure:**
9:	*P_include_* = INCLUSION (*P*(*D*), *SCG*)
10:	// **Exclusion Procedure:**
11:	*P_exclude_* = EXCLUSION (*P*(*D*), *SCG*)
12:	**IF** (*P_include_* = *P_exclude_*) **THEN**
13:	*k* ← *k* + 1
14:	*J*(*k*) ← *SCG*’ *J* value
15:	**ELSE**
16:	// **Continued Exclusion Procedure:**
17:	*P_exclude_* = EXCLUSION (*P*(*D*), *SCG*)
18:	**IF** (*P_include_* = *P_exclude_*) **THEN**
19:	*k* ← *k* + 1
20:	*J*(*k*) ← *SCG*’ *J* value
21:	**ELSE** repeat **Continue Exclusion Procedure**
22:	**END**



**Function** INCLUSION (*P*(*D*), *SCG*)	

1:	*CCGS*: candidate contrast group set
2:	*CCGS* ← *ϕ*;
3:	**FOREACH** population variable *P_i_* ∈ *P*(*D*) **DO**
4:	Compose a set of contrast pairs *CPair_i_* based on *P_i_*’s categories
5:	**FOREACH** contrasting pair (*C_i,m_*, *C_i,n_*) ∈ *CPair_i_* **DO**
6:	*SCG_temp_* ← *SCG* + ((*C_i,m_*, *C_i,n_*))
7:	Divide data *D* into (*S*_*G*1_, *S*_*G*2_) based on *SCG*_*temp*_
8:	*J*(*SCG*_*temp*_) ← CONTRAST_MINING (*S*_*G*1_, *S*_*G*2_)
9:	Add *SCG*_*temp*_ to CCGS
10:	**END**
11:	**END**
12:	Select the highest *J*-value contrast groups *SCG*_*highest*_ from CCGS
13:	*SCG* ← *SCG_highest_*
14:	Remove the population variables of *SCG*_*highest*_ from *P*(*D*)



**Function** EXCLUSION *(P(D), SCG)*	

1:	*CCGS*: candidate contrast group set
2:	*CCGS* ← *ϕ*;
3:	**FOREACH** contrasting pair (*C*_*i,m*_, *C*_*i,n*_) **DO**
4:	*SCG*_*temp*_ ← *SCG* **−** ((*C*_*i,m*_, *C*_*i,n*_))
5:	Divide data *D* into (*S*_*G*1_ *S*_*G*2_) based on *SCG*_*temp*_
6:	*J*(*SCG*_*temp*_) ← CONTRAST_MINING (*S*_*G*1_, *S*_*G*2_)
7:	Add *SCG*_*temp*_ to CCGS
8:	**END**
9:	Select the highest *J*-value contrast groups *SCG*_*highest*_ from CCGS
10:	*SCG* ← *SCG_highest_*
11:	Add the population variable of *SCG*_*highest*_ back to *P*(*D*)

As shown in Fig. 2, the *Path Expansion* process starts a root node of the cohort selection tree to perform a population variable inclusion step, which forms the first layer of nodes containing contrast subgroups with only one population variable. For example, the node *(C*_1,1_ ↔ *C*_1,2_) is to compare two subgroups based on the 1st and 2nd categories of the first selected population available. By adding one more population variable to the first layer, the second layer then contains a pair of contrast subgroups with two population variables. In the figure, the node ((*C*_1,1_ Λ *C*_2,*n*_) ↔ *(C*_1,2_ Λ *C*_2,*n*_)) is obtained by adding the *n*th category of the second selected population variable to the previous node. After an additional inclusion process, the *Path Expansion* process creates a node with ((*C*_1,1_ Λ *C*_2,*n*_ Λ *C*_3,1_) ↔ (*C*_1,2_ Λ *C*_2,*n*_ Λ *C*_3,1_)) subgroup pair at Layer 3. A later node on the path has a subpopulation of any prior node on the same path. Afterwards, a series of inclusion and exclusion processes are performed to add or remove population variables for a pair of smaller or larger cohorts. For example, by dropping the second population variable of the node ((*C*_1,1_ Λ *C*_2,*n*_ Λ *C*_3,1_) ↔ (*C*_1,2_ Λ *C*_2,*n*_ Λ *C*_3,1_)) in Fig. 2, we may achieve a better contrast subgroup pair ((*C*_1,1_ Λ *C*_3,1_) ↔ (*C*_1,2_ Λ *C*_3,1_)) with a higher *J* value compared to the previous one. As shown in Fig. 2, the new node after the exclusion ((*C*_1,1_ Λ *C*_3,1_) ↔ (*C*_1,2_ Λ *C*_3,1_)) is a duplicate of the one in the second layer. If a set of population variables has been evaluated previously or pre-defined by clinicians as trivial known subgroup pairs, the duplicated subgroup signified by a single dashed circle is pruned from the tree structure to avoid repetitive effort for contrast mining (a shell will be disabled if it aims at a target previously hit.) This pruning process in conjunction with the stopping criterion in Algorithm 1 (Line 7) ensures the algorithm will finish without entering an “oscillation” cycle.

Without searching the entire space to obtain a complete assessment of all cohorts, this floating and path expansion algorithm utilizes a floating selection process, which is less greedy and more computational feasible, to systematically evaluate and select a large number of subgroups using the metrics described in the following section.

### B. Contrast Pattern Mining

The main purpose in identifying pairs of subgroups is to discover significant contrasts that are likely to provide biomedical researchers with insights about interventions. Contrasts between a pair of subgroups could include different biomarkers between patient populations with certain phenotypic groups, as well as significant socioeconomic factors between disparity groups. To assess the differences between a pair of contrast subgroups, we extend the concepts of contrast mining methods [25] between two pre-defined subgroups to discover patterns with significant difference in prevalence. The following process describes the CONTRAST_MINING() function as listed in the INCLUSION and EXCLUSION pseudo codes. We use support and growth rates [25] for the initial evaluations of contrast patterns frequently appearing in one group but seldom in the other group. Given a data collection (*D*) of all patients and a collection of *n* measurable variables *M* = (*m*_1_, *m*_2_, … , *m*_*n*_} discussed in Section III, a patient’s record *r* ∈ *D* contains some instances of the subset of measurement variables. The total number of records in *D* is noted as |*D*|. A pattern appearing in *r* is a set of categories of several measurable variables, such as *p* = (*m*_*i*,*k*_, … , *m*_*j,l*_}, where *m*_*i,k*_ is a category of measurable variable *m*_*i*_ and *m_j,l_* is a category of measurable variable *m_j_*. The support of a pattern *p* from *D* is the ratio of the number of records containing *p* to the total number of records in the collection, denoted as

(2) $$Support(p,D)=\frac{\left|\langle D,p\rangle \right|}{\left|D\right|}$$

Given two exclusive subgroups *S*_*G*1_ and *S*_*G*2_, a contrast pattern *cp* is the pattern whose support differs significantly between the two subgroups. If the support of *cp* in *S*_*G*1_ is *s*_1_ and the support of *cp* in *S*_*G*2_ is *s*_2_, the degree of its differences can be represented by growth defined as follows:

(3) $$Growth\left(cp,S_{G1},S_{G2}\right)=\frac{\mathrm{Max}\left\{s_{1},s_{2}\right\}}{\mathrm{Min}\left\{s_{1},s_{2}\right\}}$$

The range of growth is [1, +∞). The bigger the differences, the greater the growth. To normalize the growth value, we extend the tanh function [40].

(4) $$\begin{array}{c} {\mathrm{Growth}}_{Norm}=\tanh\left(\frac{\mathrm{Growth}\left(cp,S_{G1},S_{G2}\right)}{{\mathrm{Growth}}_{\max}}\right)\\ {}*{\mathrm{Growth}}_{\max}, \end{array}$$

where *Growth*_max_ is the estimated maximal growth rate of a contrast pattern appearing in random contrast subgroup selection or a user-defined upper bound. After the normalization, the growth value is in the range [0, *Growth*_max_).

Each contrast pattern between the pair of subgroups has to be frequent in at least one of the subgroups and its prevalence difference must be significant. Let *α* and *β* be the thresholds for support and growth rate, respectively. To ensure that a *cp* is frequent and has a significant prevalence difference between a pair of subgroups, the condition (*Support*(*cp, S*_*G*1_) ≥ *α* OR *Support*(*cp, S*_*G*2_) ≥ *α*) AND (*Growth*(*cp, S*_*G*1_, *S*_*G*2_) ≥ *β*) must be held. Applying this condition will identify two sets of contrast patterns *CP*^1^ and *CP*^2^ for the selected pair of subgroups *S*_*G*1_ and *S*_*G*2_. In addition, for each contrast pattern *cp*_*n*_ with multiple measurable variables, the subset of the pattern *cp_i_* ⊆ *cp*_*n*_ will be kept when *Growth*(*cp*_*i*_, *S*_*G*1_, *S*_*G*2_) − *Growth*(*cp*_*n*_, *S*_*G*1_, *S*_*G*2_) > 0. Those selected contrast patterns are called effective contrast patterns and are utilized to evaluate each pair of subgroups during the floating and path expansion procedure discussed in Section IV.A. The selection of an *α* value is based on the clinical application. For example, in a population health study, the appropriate value of *α* should consider sufficient size of population affected by the pattern; while in a rare disease study, the value of *α* could be as low as 0.05% to ensure the target populations with that pattern are not neglected in the process. When the population size drops to a certain number, a high *α* value should be applied to ensure the contrast patterns are commonly shared by the majority of patients in the small-sized subpopulation. The selection of *β* value is normally greater than 2.0 to ensure that the extracted contrast patterns appear at least twice as often in one subpopulation compared to the other.

### C. Subgroup Prioritization Using J-Value

The outcome of the path expansion algorithm (Section IV.A) results in hundreds or even thousands of candidate subgroup pairs. To prioritize the subgroup pairs from the candidate pool for clinical trials or future studies, we evaluate the aggregated contributions of the extracted contrast patterns within each pair of subgroups (Section IV.B) based on two factors: (1) number of contrast patterns and (2) significance of those patterns.

To evaluate the overall quality of a set of contrast patterns that are significantly more frequent in one subgroup than in another subgroup, we use the quantitative indicator *J* value inspired by the *g-index*, which is commonly used to evaluate the productivity of a scholar [41]. If a researcher has published a set of articles (*cp* patterns), the g-index is measured by ranking them in decreasing order based on their citations (*growth rate* for each *cp* pattern). If a contrast subgroup has a set of *cp* patterns (articles), the *J* value is measured by ranking them in decreasing order based on their growth rate and then by taking the largest number such that the top *J cp* patterns (top *g* articles) cumulatively received at least *J*^2^ (*g*^*2*^ citations) scores. The *J* value is defined as follows:

(5) $$J^{2}\leq \sum_{i\leq J}{\mathrm{Growth}}_{Norm}\left(cp_{i},S_{G1},S_{G2}\right)$$

In the biomedical area, researches could focus on small subpopulations to target treatment for a small group of patients for precision medicine, such as one with rare diseases (1 of 2,000 or less), or to study a population for health disparities between urban and rural groups (tens of thousands of subjects). To consider this population size factor, we applied the concept of Bayesian Average [42] that will allow us to set priority based on population size. For a contrast group with population size *n* and an original evaluation value of *J*_*ori*_, the size-modified *J* value is defined as:

(6) $$J_{\mathrm{size}-\text{modified}}=\frac{N*J_{\mathrm{ori}}+M*\bar{J}}{N+M},$$

where $N=\left\{n,\frac{1}{n}\right\}$, *N* = *n* when a larger population is preferred and $N=\frac{1}{n}$ when a smaller population is preferred. $\bar{J}$ is the average evaluation value and *M* is the average population size of randomly picked contrast subgroups prior to the path expansion process. Given *k* random selected contrast subgroups, $\bar{J}=\sum_{i=1}^{k}J_{i}/k$ and the *M* = (*s*_1_ + ⋯ + *s*_*k*_)/*k*, where *J*_1_, … , *J*_*k*_ is the original evaluation value *J*_*ori*_ of the random selected contrast subgroups and *s*_1_, … , *s*_*k*_ are their population sizes.

At the final subgroup prioritization step, all candidate contrast subgroups are ranked based on their *J* values.

## V. Distributed Computing Algorithms

**Fig. 3.**
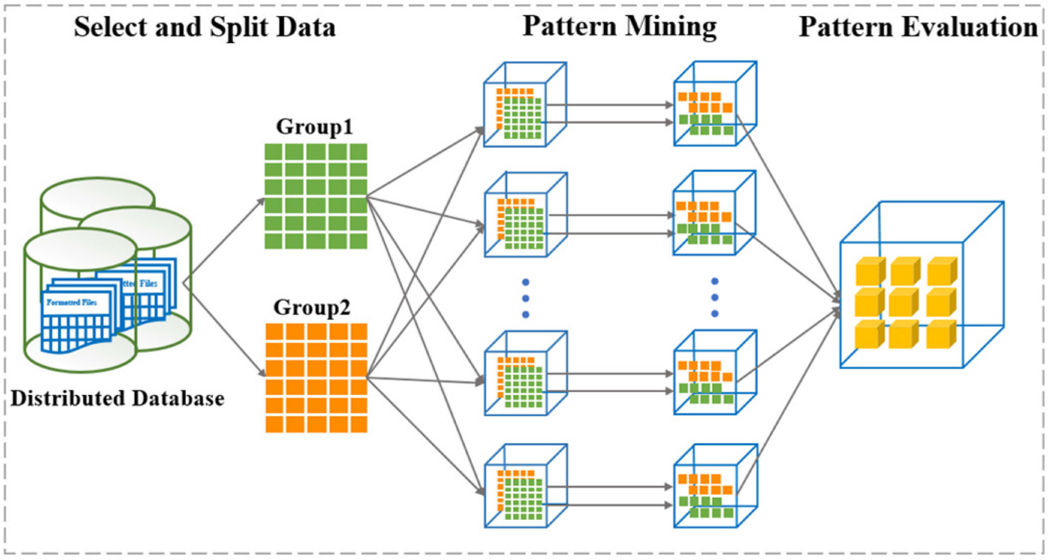
Distributed pattern mining for a contrast subgroup using an Apache Spark high performance computing environment.

Due to the combinatory challenges encountered when exploring subgroups from all population variables (*P*) (tens of thousands of potential subgroup pairs) and extracting contrast patterns from a large number of measurement variables (*M*) (millions of potential patterns), we utilize a distributed computing framework for this project. There are two computationally expensive procedures which can be accelerated: (1) the Floating Path Expansion as shown in Fig. 2, and (2) the Effective Contrast Pattern Extraction for detection used in *J* value calculation. Our method is implemented in Apache Spark [43] which allows us to take advantage of high throughput computing resources. As depicted in Fig. 3, to find effective contrast patterns, we first apply the FP-Growth algorithm [44] which is proven efficient using an elegant prefix tree to mine the frequent patterns in a selected contrast subgroup with two groups *S*_*G*1_ and *S*_*G*2_. We then aggregate these patterns to calculate their growth rates. The patterns that satisfy the conditions in Section IV.B are selected as candidates and used to calculate the *J* value.

By applying Floating Contrast Subgroup Selection (Algorithm 1), the contrast subgroup with the highest *J* value is discovered first, and then the Guided Cascading Shotgun Approach is used for Path Expansion process to obtain the top-K results with high *J* values. This process is implemented in a distributed in-memory computing environment to load paths and their mined patterns in large-memory clusters.

## VI. Experiments

We evaluate the deep exploratory mining method using both synthesized and autism research [45] datasets for cohort discovery and ranking. Because discovery from real datasets is challenging to assess other than by validating it through existing literature, we used a synthesized data set to evaluate the coverage of randomly pre-defined cohorts using a range of expansion factors and computational resource needs. For the autism research dataset, the assessment is mainly on the discovery of new findings, which are considered novel based on the autism literature.

### A. Synthetic Data – Cohort Coverage and Computing Resources Assessments

**Fig. 4.**
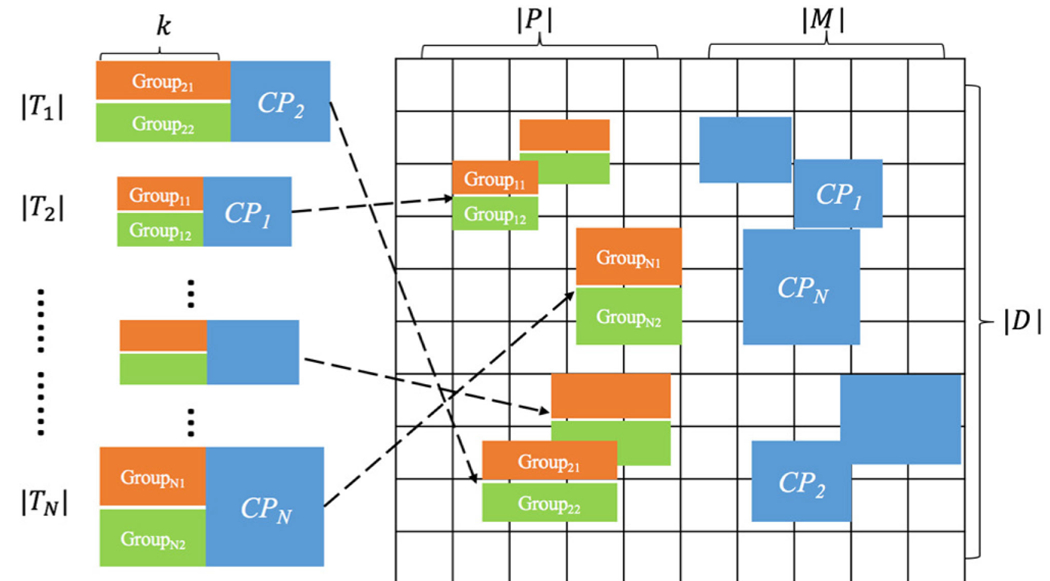
The generation of a synthesized dataset containing subgroup pairs where contrast patterns have various overlapping factors in the measurement (*M*) space with varying length of patterns. There are *N/k* subgroup pairs for lengths from 1 to *k* randomly assigned to the dataset.

To explain the setting of the synthesized data set, we use Fig. 4 to pictorially describe the concept of the data creation process. The synthesized dataset, with size |*D*|, contains |*P*| population variables and |*M*| measurement variables. Each population variable (e.g., age) has *P*_*c*_ category values (e.g., age groups for a certain intervention) and each measurement variable (e.g., lab test values) has *M*_*c*_ categories (e.g., low, normal, and high).

We first created *N* pairs of pre-defined subgroups as artificial cohorts with significant contrasts from the measurements between each pair. The length (inclusion criterion) of pairs of subgroups varied from 1 to *k* population variables. In total, we created *N/k* subgroup pairs for each length. The sample size of a contrast subgroup was |*T*| using a uniform distribution. We assigned contrast patterns to each pair of subgroups to ensure that those subgroups contain pre-defined high contrast patterns. Each contrast pattern was frequent in at least one of the subgroup pairs with a significant growth rate. By following the *Apriori* property of the association rule mining process, a short pattern had a higher or equal support value (frequency) than its superset. All subgroup pairs were randomly formed and various measurement associations with different lengths were randomly assigned to each subgroup pair. Other measurements were then filled with random categories using the Gaussian distribution. In addition, to test the robustness of our methods, we intentionally assigned various levels of overlaps between pairs of subgroups with an expectation to increase the difficulty of the deep exploratory mining in identifying overlapped subgroups.

In our synthesized data creation process, we set |*D*| = 10^6^, |*M*| = 100, *P*_*c*_ = *M*_*c*_ = 10, *k* = 5 and |*T*| ~ *U*[0.01 × |*D*|, 0.1 × |*D*|]. The maximal subgroup length *k* = 5 was determined based on our empirical observations from real biomedical applications in cohort studies. We defined the subgroup sample size |*T*| that varied from 1% to 10% of the total data size. In addition, we chose four pools of population variables |*P*| ∈ {5, 10, 15, 20} to test the effectiveness of the method. For each pool, we set the number of subgroup pairs *N* = 10, 20, 30, and 40.

To test whether the deep exploratory mining method is able to identify the majority of the artificial cohorts and to assess the necessary computational resources to achieve the goal, we performed the subgroup cohort discovery method on a collection of synthesized datasets with a different number of population pools using a set of expansion factors ranging from 5% to 20%. The Support threshold *α* is set as 0.5 and Growth threshold *β* is set as 2 in the experiment. These expansion factors from the “*Guided Cascading Shotgun*” approach (Section IV.A) were used to evaluate how “deep” the approach should explore to ensure a certain coverage of the artificial cohorts. For each pool of population variables, the deep exploratory mining experiment was repeated five times and the average of the coverages was calculated. Ideally, the deep exploratory mining process should be able to identify all cohorts with a small expansion factor.

**Fig. 5.**
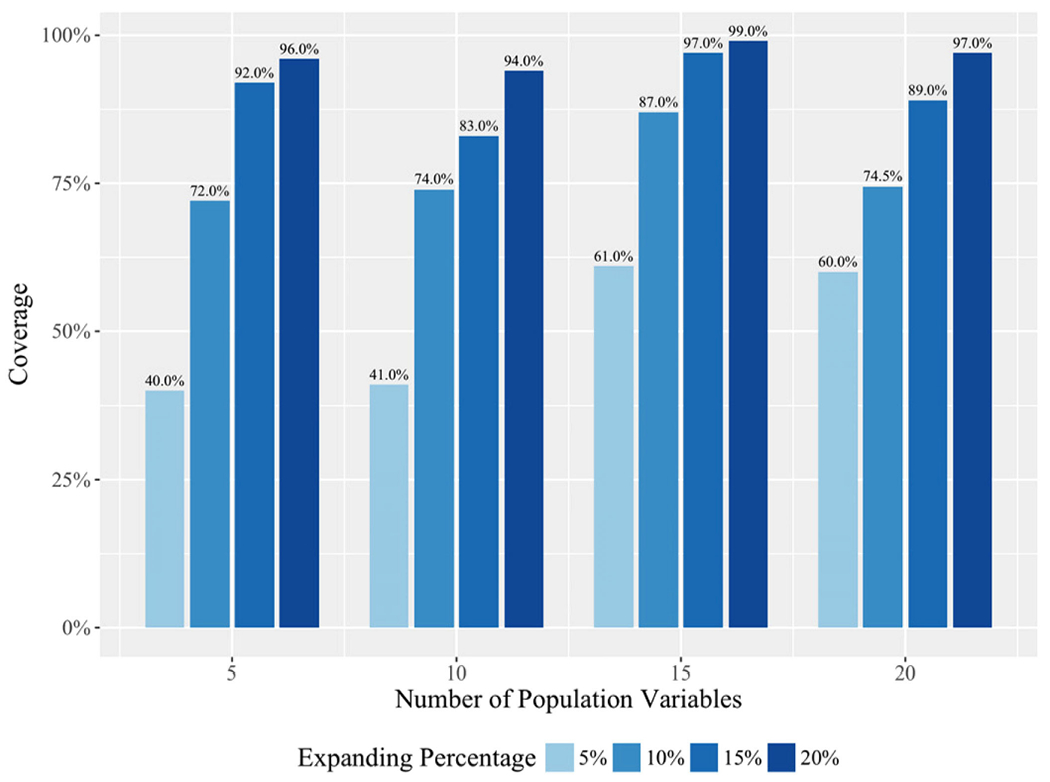
The coverage of all artificial cohorts discovered by the algorithm on the synthesized data. Each synthesized dataset has one million records. Synthesized data with the population variable numbers range from 5 to 20. The expanding percentage ranges from 5% to 20%.

As shown in Fig. 5, the coverages were improved when the expansion factors increased, as expected. However,100% coverage was not reached even with a 20% expansion factor which consumed a significant amount of computing resources. Manually inspecting the cohorts newly discovered by the method but not in the pre-defined sets, we observed that the “*Guided Cascading Shotgun*” approach discovered other potential paths, which had better contrasts than some of the artificial cohorts. When the exploration went deeper – for 10% and 20% expansion factors, the coverages were above 72%, and 94%, respectively. For this synthesized data set, an expansion factor larger than 20% may not provide sufficient economic benefits to cover those “left out” subgroup cohorts that are not so meaningful as the newly discovered ones.

**Fig. 6.**
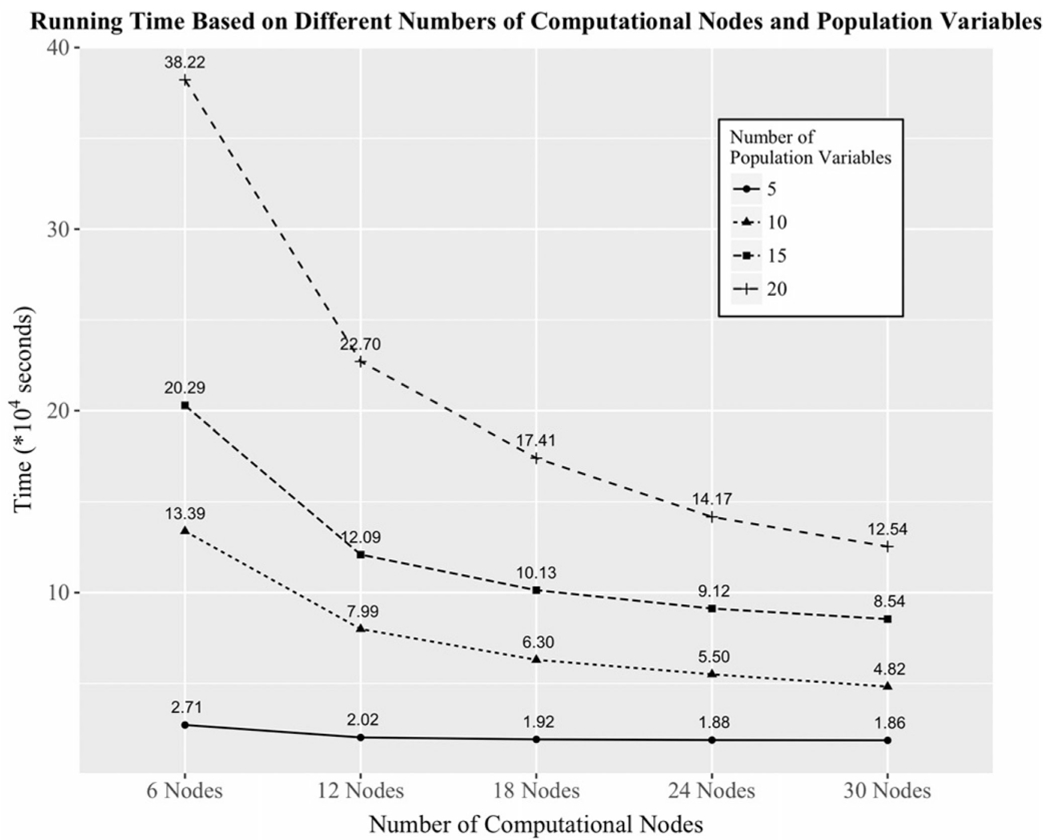
The running time of different numbers of population variables with expanding factor equals to 20% based on 6, 12, 18, 24, and 30 computational nodes.

In Fig. 6, we compared the running times of the different pools of population variables with a 20% expansion factor using 6, 12, 18, 24, and 30 computational nodes. Each node was allocated an Intel Xeon CPU E5-2670 v3 @ 2.30 GHz with a 21-core processor and 105 GB RAM for the computing time study. Fig. 6 shows that the running time of different numbers of population variables had a noticeable gap because the search space increases when more population variables were added to the experiments. A larger number of population variables clearly requires more computing nodes to reduce the extra running time due to the combinatorial search space. From the figure, the running times, using six computing nodes, between the data sets with 5- and 20-population variables are about 7.53 hours and 4.42 days, respectively. When the number of computing nodes was doubled, the running time for data set with 5-population variables reduced by 25% (1.92 hours) while the running time for a data set with 20-population variables significantly reduced by 40.6% (43.11 hours). As shown in the figure below, the datasets with small number of population variables do not require extensive computing resource due to relatively much smaller search space than those with large number population variables. Therefore, adding more computational nodes does not significantly reduce the running time. It is worth noting that the number of samples in the subgroup cohorts insignificantly affects the running time compared to the number of population variables and expansion factors. Figs. 5 and 6 provide a general assessment for researchers seeking to allocate appropriate computing resources based on number of population variables and coverage expectations.

While there is no method that provides precisely the same function of our approach for a fair comparison, in Supplement 4.A, we compared the results of our method with hierarchical clustering [46] and network analysis [47] which are “bottom-up” approaches using measurable variables to form clusters where common population variables are considered novel cohorts. We used a subset of the synthesized data set to evaluate coverage of pre-defined subgroups. Results are reported in Supplement 4.A.

### B. Autism Data Set – Novel Discovery Assessment

Autism Spectrum Disorder (ASD) is a developmental disorder which results in lifelong impairments and disability in social skills, repetitive behaviors, and speech and communication issues [48]. About 1 in 59 children are diagnosed with some form of ASD according to the CDC’s Autism and Developmental Disabilities Monitoring (ADDM) Network [49]. ASD is comprised of many different subgroups both genetically and phenotypically, and there is an urgent need to subcategorize ASD patients and tailor treatments for each patient [36], [50], [51].

In our real case study, we use the Simons Foundation Autism Research Initiative (SFARI) Simon’s Simplex Collection (SSC) [45]. The data contains 2591 families with exactly one child diagnosed with autism (proband) while the parents and siblings are unaffected. The data contains demographic information, family history and several behavior assessments and diagnostic aids as phenotype data. Also, the genotype data is collected from all probands and their 7605 unaffected family members. In this experiment, we chose 15 phenotype features as population variables consisting of cumulative scores, IQ scores, language development, emotion or behavior problems, assessment subscales, developmental milestones and physical attributes, and pre-selected 10,000 Single Nucleotide Polymorphisms (SNPs) by utilizing genome-wide SNP prioritization to preliminarily discover novel associations related to Autism. Those SNPs are used as measurement variables to differentiate subgroup cohorts. By performing the deep exploratory data mining method with a 20% expanding factor, we discovered 142 contrast subgroups. Running times were 1.58 days, 20.28 hours, 14.31 hours, 11.64 hours, and 9.67 hours using 6, 12, 18, 24, and 30 computational nodes, respectively, with the same five settings of computational resource used in the synthesized data set.

From the discovered subgroup cohorts, we separated and ranked contrast subgroups for single-, double-, and triple-population variable settings and picked the top two most contrasted subgroups from each of them as listed in Table I. We used Fisher’s exact test [52] to assess statistical significance of the identified genes and listed their P values in Supplement 1. We listed the top 10 subgroup pairs ranked by their *J* values for each population variable setting in Supplement 3. To empirically prove that the significant genes are unique on a specific side of a subgroup pair, we treat the family members as outgroup and check the gene significance by comparing with the outgroup. We searched those significant genes in AutDB, an evolving database for the autism research community [53], and PubMed abstracts of autism related publications. From all discovered genes or gene combinations in the top 20 subgroup cohorts, 11.57% of 415 relevant genes are in AutDB, nearly 20.72% were identified through the PubMed search, and the remaining genes were considered novel. We then further studied each contrast subgroup pair to find whether there are any publications to support those contrast subgroups and significant genes. Table I lists six subgroup pairs for cohorts with single-, double-, and triple-population variables, using support threshold as 0.2 and growth rates 2.5, 3.0, and 4.0, respectively. In the table, “No. of Discovered Genes” reports the number of distinct genes identified by the algorithms, “No. of Genes in AutDB” lists the number of identified genes is in the AutDB, and “No. of PubMed Articles” lists the numbers of articles studied the selected population variables of a pair of subgroups without restriction of quantifiers, such “Low,” “Mid,” or “High.”

#### Single-Population-Variable Subgroup Pairs:

Our algorithm identified two contrast subgroups [Low SSC Full Scale IQ] versus [High SSC Full Scale IQ] with five significant genes discovered using a 2.5 growth rate threshold. One of them is listed in the AutDB. 2242 articles meet the search criterion “(autism OR asd) AND (IQ OR “intelligence quotient”)” from PubMed. While there are 104 articles having genetic discussions with the subgroup (low IQ and high IQ), there is no article discussing the relationship between IQ with any of the five discovered genes. The five genes are considered novel for this pair of subgroups. A further study of the contrast patterns showed that gene combinations (SIRT2, CSGALNACT1) (support: 0.209 vs 0.075) and (ARHGAP24, ATP10B) (support: 0.214 vs 0.078) appear 2.7 more times in the [Low SSC Full Scale IQ] subgroup than the [High SSC Full Scale IQ] subgroup. The gene ARHGAP24 is known to be associated with ASD based on the AutDB and PubMed search while SIRT2, CSGALNACT1, ATP10B are new genes discovered by our methods. The mined results will provide the autism community potential directions to conduct in-depth study for the topic related to high or low intelligence quotient (IQ), such as Chiocchetti AG *et al*.’s investigation of the functional common variants of glutamatergic genes between cohorts of lower (IQ ≤ 70; LIQ) and higher intellectual ability (IQ > 70; HIQ) cohorts [54].

In the table, we also report the statistics for another pair of single-population-variable subgroup cohorts related to “Normal/Late to Speak Sentences” for language impairment, which is an established topic in the autism research community [55], [56]. We found that the co-occurrence of genes (PIEZO1, ACSS3) (support: 0.078 vs 0.237) is 3.04 times greater in the language impairment subgroup (Late to Speak Sentences) compared to the normal one (Normal to Speak Sentences). Gene combination (SCN5A, ACSS3) (support: 0.078 vs 0.217) is 2.78 times more prevalent in the language impairment subgroup while (EDARADD, PPP2R2B) (support: 0.240 vs 0.092) is 2.6 times more prevalent in the unaffected group.

#### Double-Population-Variable Subgroup Pairs:

Our algorithm identified a pair of contrast subgroups [Mid RBS-R Overall Score AND Low CBCL6 Social Score] versus [Low RBS-R Overall Score AND Low CBCL6 Social Score] with 44 significant genes discovered using a 3.0 growth rate threshold. Three of them are listed in the AutDB. 898 articles were retrieved using a search criterion, listed in Supplement 2, from the PubMed using lexical variations of the double population variables. Among them, 179 articles had some genetic discussions and four papers mentioned discovered genes-GATA3, KIRREL3, CLSTN2 are associated with this pair of subgroups [57]–[60]. We found that gene combination (KIRREL3, SRGAP3) (support: 0.208 vs 0.052) is 4.00 times more prevalent in those with a mid-range Repetitive Behaviors Scale – Revised scores (RBS-R) and who have a low CBCL6 social scores as compared to the group, which has a low Repetitive Behaviors Scale – Revised scores (RBS-R) but also has a low CBCL6 social scores. Gene combination (PTPRF, SRGAP3) (support: 0.327 vs 0.078) is 4.19 times more prevalent in the group with mid-range Repetitive Behaviors Scale – Revised scores (RBS-R) while (CLSTN2, WDFY4) (support: 0.054 vs 0.208) is 3.81 times more prevalent in the group with low Repetitive Behaviors Scale – Revised scores (RBS-R). Genes KIRREL3, SRGAP3, CLSTN2 and WDFY4 are associated with autism in the PubMed Search, and gene PTPRF has not been reported in the autism literature so far. Also, gene KIRREL3 is associated with the accessory olfactory system, which controls social, sexual interactions and is related to repetitive behaviors in mice [60], [61], and is a candidate gene for social and language delay in autism patients [62]. Gene SRGAP3 is also a risk gene for schizophrenia and associated with impaired social behavior [63]. Gene CLSTN2 is also suggested for a possible role in the psychopathological mechanisms of autism [59].

In the same group of Table I, we also report the statistics for another pair of double-population-variable subgroup cohorts relating “Low/High ABC III Stereotypy Scale” and “Late to Use Words.” We found that the gene combination (GRIN2B, ASB1) (support: 0.053 vs 0.208) appeared to be 3.94 times more prevalent in autistic patients scoring a high on the ABC III Stereotype scale (Aberrant Behavior Checklist Stereotypic Behavior) and who are late to use words as compared to the group with a low ABC III Stereotype scale scores and that is also late to use words. GRIN2B is shown in AutDB and ASB1 and is known to be autism related through PubMed Search, where GRIN2B is reported to be associated with verbal fluency and linguistic processes [64]. However, none of the 44 significant genes were reported in the literature related to the specific subgroup populations.

#### Triple-Population-Variable Subgroup Pairs:

Our algorithm identified a pair of contrast subgroups [Mid Vineland II Daily Living AND High Height Z Score AND High ADIR C Total] versus [High Vineland II Daily Living AND High Height Z Score AND High ADIR C Total] with 22 significant genes discovered using a 4.0 growth rate threshold. Four of them are listed in the AutDB. However, no article meets the search criterion, listed in Supplement 2, from the PubMed using lexical variations of the triple population variables.

The co-occurrence of genes (KCNQ4, KCNH1) (support: 0.213 vs 0.019) appears 11.52 times more in the group which has “Mid-range Vineland II daily living scores, a high height score and a high ADIR C Total Score” than the group which has “High Vineland II daily living scores, a high height score and a high ADIR C Total Score.” Genes combination (PPM1E, TET2) (support: 0.265 vs 0.037) is 7.15 times more frequent in the group with a mid-range Vineland daily living score. Gene TET2 is associated with autism in the PubMed Search while genes KCNQ4, KCNH1 and PPM1E are considered new discoveries which are not reported in the autism literature yet.

In the same group of the table, we also report the statistics for another pair of triple-population-variable subgroup cohorts related to “Med/High CBCL6 Rule Breaking Score,” “Low CBCL6 Activities Score,” and “High SRS-P Total Score.” Co-occurrence of genes (FHIT, ZNF578) (support: 0.206 vs 0.034) appears 6.08 times as much in the group with mid-range rule breaking scores than in the group with high rule breaking scores. The gene combination (CNTN5, KIAA1211L) (support: 0.276 vs 0.051) appears 5.43 times more in the group with mid-range rule breaking scores. These new findings will provide suggestions to the autism research community to focus on more targeted subgroup cohorts which were not investigated previously.

To conduct a comparison of unsupervised clustering methods for cohort discovery, we applied the hierarchical clustering and network analysis on the full Autism data set and the results are discussed in Supplement 4.B.

## VII. Conclusion

In almost all biomedical research activities, finding small homogenous subgroups within a large heterogeneous population is a critical process for hypothesis formulation. Patient sample heterogeneity plagues efforts to target individualized treatments by masking critical individual and subgroup variation within samples. Genomic variation, epigenetic influences, molecular metabolic factors, and demographic and social factors differ widely within patient populations and can be important indicators of treatment response. The success of identifying optimal subpopulations is expected to result in much more promising findings for tailoring treatment than simply looking at the population as a whole for precision health research.

In this paper, we present a novel deep exploratory mining framework for subgroup cohort discovery. This framework consists of a floating and path expansion process, contrast pattern mining, and subgroup prioritization using *J*-value. This work demonstrates a robust automatic cohort prioritization process by strategically exploring multi-dimensional population variables to form meaningful subgroups, which have explainable and highly contrasted genotypic/phenotypic patterns that may benefit from intervention. We implemented the framework and deployed it in a distributed computing environment to ensure an efficient mining process. A series of computational experiments was conducted to assess the resource needs for various dimensions, such as complexity of the data (number of potential population variables) and availability of computing power (number of nodes). To test the capability of the work, we perform computational evaluation on both synthetic and autism datasets. The ranked cohorts from the synthesized data set show the high percentage of coverage of pre-set subgroups, as well as novel findings of subgroups that were identified only by the framework with patterns having better contrasts than those in the pre-set data. In addition, the results from the autism data set demonstrate novel discoveries of genes that are new to the autism research community [36]. The mined subgroup cohorts and relevant genetic patterns will provide the community with data-driven and statistically tested knowledge to develop hypotheses for more in-depth wet lab studies or clinical trials.

While categorization in the data mapping process makes the findings explainable, it poses limitation related to the loss of information granularity. Applying imputation to estimate missing values in the autism study could bring bias to the data set. Our future works are to develop a pattern mining module to handle continues measurable variables using fuzzy thresholding [65] to avoid artificial crisp partitioning in categorization. Moreover, we plan to embed the cost and impact on intervention development, such as drug repositioning [66], in the *J* value calculation, to tailor meaningful cohorts of patients. In addition to applications in genomics, we will extend our work to perform cohort discovery from electronic health record, medical images, and other biomedical data modalities.

This framework will provide the broad biomedical research community with a means to develop strategies to identify homogeneous subgroups within heterogeneous populations prior to conducting costly bench experiments or clinical trials. It has the potential to enable targeted treatments to improve outcomes, reduce costs, and minimize morbidity associated with misdirected interventions.

## Supplementary Material

Supplement 1

Supplement 2

Supplement 3

Supplement 4

## Figures and Tables

**TABLE I:** Rated Contrast Subgroups and Ratio of Published Significant Genes

Subgroup 1 ^a^			Subgroup 2		No. of Discovered Genes	No. of Discovered Genes also in AutDB ^b^	No. of PubMed Articles
		
Population Variable(s)	Cohort Size		Population Variable(s)	Cohort Size	Number	Number	Number
Low SSC Full Scale IQ	459	vs	High SSC Full Scale IQ	373	5	1	2242
Normal to Speak Sentences	346	vs	Late to Speak Sentences	304	16	3	5130

Mid RBS-R Overall Score **AND** Low CBCL6 Social Score	202	vs	Low RBS-R Overall Score **AND** Low CBCL6 Social Score	77	44	6	898
Low ABC III Stereotypy Scale **AND** Late to Use Words	171	vs	High ABC III Stereotypy Scale **AND** Late to Use Words	159	18	2	452

Mid Vineland II Daily Living **AND** High Height Z Score **AND** High ADIR C Total	253	vs	High Vineland II Daily Living **AND** High Height Z Score **AND** High ADIR C Total	54	22	4	0
Mid CBCL6 Rule Breaking Score **AND** Low CBCL6 Activities Score **AND** High SRS-P Total Score	228	vs	High CBCL6 Rule Breaking Score **AND** Low CBCL6 Activities Score **AND** High SRS-P Total Score	59	25	4	0

SSC Full Scale IQ = Simons Simplex Complex Full Scale IQ, RBS-R = Repetitive Behaviors Scale-Revised, CBCL6 = Child Behavior Checklist for ages 6-18, ABC III = Aberrant Behavior Checklist-Stereotype Scale, Vineland II Daily Living = Vineland Adaptive Behavior Scales-Second Edition in Daily Living domain, ADIR C Total = Autism Diagnostic Interview-Revised (ADI-R)-Restricted, Repetitive, and Stereotyped Patterns of Behavior total score, SRS-P = Social Responsiveness Scale – Parent Report.

Details about significant genes are in the Supplement 1.
