# Supplementary material for: Exploratory Data Mining for Subgroup Cohort Discoveries and Prioritization
Source: IEEE J Biomed Health Inform. Author manuscript; Available in PMC 2022 Aug 1. (PMC9341221; doi:10.1109/JBHI.2019.2939149)
Supplement: Supplement 1 [file NIHMS1822002-supplement-Supplement_1.pdf]

Supplement 1

This supplement document contains the discovered significant genes of six contrast subgroups reported in the main paper. Fisher's exact test is used to calculate exact P value of each gene. The Fisher's exact test were employed when the sample sizes are small.

Contrast Subgroup 1:

Subgroup1: Low SSC Full Scale IQ
Subgroup2: High SSC Full Scale IQ

SNP_ID1	Gene Symbol1	In_AutDB?	SNP_ID2	Gene Symbol2	In_AutDB?	Group1 Size	Support1	Growth1	Goup2 Size	Support2	Growth2	Chi Square between two subgroups	Chi Square compared to family members	P-Value
rs892034	SIRT2	NOTIN	rs12676278	CSGALNACT1	NOTIN	459	0.209150327	2.786181139	373	0.075067024	0.358914209	29.47598785	14.98825234	<0.0001
rs345325	ARHGAP24	IN_AutDB	rs62391601	ATP10B	NOTIN	459	0.213507625	2.746149801	373	0.077747989	0.364146195	28.28177407	21.64123513	<0.0001
rs62391601	ATP10B	NOTIN	rs1172459	RPL13AP5	NOTIN	459	0.222222222	2.511784512	373	0.08847185	0.398123324	26.10620736	28.47049271	<0.0001

Contrast Subgroup 2:

Subgroup1: Normal to Speak Sentences
Subgroup2: Late to Speak Sentences

SNP_ID1	Gene Symbol1	In_AutDB?	SNP_ID2	Gene Symbol2	In_AutDB?	Group1 Size	Support1	Growth1	Goup2 Size	Support2	Growth2	Chi Square between two subgroups	Chi Square compared to family members	P-Value
rs11114679	ACSS3	NOTIN	rs1172459	RPL13AP5	NOTIN	346	0.083815029	0.386057103	304	0.217105263	2.590290381	21.98220021	22.26791219	< 0.00001
rs12915695	CHRNA7	IN_AutDB	rs11114679	ACSS3	NOTIN	346	0.095375723	0.391813779	304	0.243421053	2.552232855	24.72571548	27.31086776	< 0.00001
rs601403	EDARADD	NOTIN	rs580633	PPP2R2B	NOTIN	346	0.239884393	2.604459125	304	0.092105263	0.38395688	23.92346955	10.90043137	< 0.00001
rs10201323	EFHD1	NOTIN	rs11114679	ACSS3	NOTIN	346	0.086705202	0.387623257	304	0.223684211	2.579824561	22.6562944	27.18373117	< 0.00001
rs7969671	HPD	NOTIN	rs11114679	ACSS3	NOTIN	346	0.083815029	0.391996443	304	0.213815789	2.551043557	21.06970695	26.00443181	< 0.00001
rs11125022	KLHL29	NOTIN	rs11114679	ACSS3	NOTIN	346	0.095375723	0.397181091	304	0.240131579	2.517743222	23.79522078	21.96870656	< 0.00001
rs7201610	PIEZO1	NOTIN	rs11114679	ACSS3	NOTIN	346	0.078034682	0.329479769	304	0.236842105	3.035087719	30.39146578	27.15491481	< 0.00001
rs28409216	PLXDC1	NOTIN	rs11114679	ACSS3	NOTIN	346	0.086705202	0.387623257	304	0.223684211	2.579824561	22.6562944	18.30964655	< 0.00001
rs10416031	POLR2E	NOTIN	rs11114679	ACSS3	NOTIN	346	0.078034682	0.382621667	304	0.203947368	2.613547758	20.65706437	10.3038602	< 0.00001
rs4712850	RIPOR2	NOTIN	rs62332760	TET2	IN_AutDB	346	0.080924855	0.39679284	304	0.203947368	2.520206767	19.51226329	14.81440777	< 0.00001
rs3922844	SCN5A	IN_AutDB	rs11114679	ACSS3	NOTIN	346	0.078034682	0.359432475	304	0.217105263	2.782163743	24.40505596	27.19300715	< 0.00001
rs12352790	UNC13B	NOTIN	rs11114679	ACSS3	NOTIN	346	0.078034682	0.364962205	304	0.213815789	2.740009747	23.44907982	21.85098996	< 0.00001
rs10982240	WHRN	NOTIN	rs11114679	ACSS3	NOTIN	346	0.086705202	0.361073719	304	0.240131579	2.769517544	27.42632797	33.33499963	< 0.00001

Contrast Subgroup 3:

Subgroup1: Mid RBS-R Overall Score AND Low CBCL6 Social Score
Subgroup2: Low RBS-R Overall Score AND Low CBCL6 Social Score

SNP_ID1	Gene Symbol1	In_AutDB?	SNP_ID2	Gene Symbol2	In_AutDB?	Group1 Size	Support1	Growth1	Goup2 Size	Support2	Growth2	Chi Square between two subgroups	Chi Square compared to family members	P-Value
rs2509151	ANO1	NOTIN	rs2399867	CAMK1D	NOTIN	202	0.064356436	0.309715347	77	0.207792208	3.228771229	10.82798044	7.577005914	0.0015
rs62391601	ATP10B	NOTIN	rs1580218	MIR4300HG	NOTIN	202	0.074257426	0.317656766	77	0.233766234	3.148051948	12.11955972	10.99850566	0.0006
rs2306422	CYBA	NOTIN	rs10191109	EFHD1	NOTIN	202	0.064356436	0.309715347	77	0.207792208	3.228771229	10.82798044	8.874722524	0.0015
rs1253625	EDARADD	NOTIN	rs13134636	LINC02511	NOTIN	202	0.207920792	4.002475248	77	0.051948052	0.249845393	8.753478818	10.04016371	0.0011
rs10191109	EFHD1	NOTIN	rs3909150	DPY19L2P1	NOTIN	202	0.069306931	0.266831683	77	0.25974026	3.747680891	15.1791378	10.78071849	<0.0001
rs3847417	GATA3	NOTIN	rs181207181	FAM86KP	NOTIN	202	0.064356436	0.309715347	77	0.207792208	3.228771229	10.82798044	7.100116634	0.0015
rs35762204	IGHVIII-67-3	NOTIN	rs9396927	OFCC1	NOTIN	202	0.247524752	3.811881188	77	0.064935065	0.262337662	12.44170508	8.758076275	0.0004
rs4660463	KCNQ4	NOTIN	rs9390068	AIG1	NOTIN	202	0.074257426	0.300937989	77	0.246753247	3.322943723	13.93642305	6.978095627	0.0003
rs4660463	KCNQ4	NOTIN	rs2109793	DPF3	NOTIN	202	0.103960396	0.32019802	77	0.324675325	3.123067409	18.15915124	9.88480803	0.0001
rs4660463	KCNQ4	NOTIN	rs2706703	PITPNC1	NOTIN	202	0.054455446	0.246651136	77	0.220779221	4.054309327	15.29513036	11.37334971	0.0002
rs4660463	KCNQ4	NOTIN	rs2306422	CYBA	NOTIN	202	0.064356436	0.291496797	77	0.220779221	3.430569431	12.63654458	9.141690732	0.0004
rs4660463	KCNQ4	NOTIN	rs12915215	UNC13C	NOTIN	202	0.069306931	0.313919627	77	0.220779221	3.185528757	11.46780027	9.434340533	0.001
rs4660463	KCNQ4	NOTIN	rs2945389	KSR1	NOTIN	202	0.069306931	0.313919627	77	0.220779221	3.185528757	11.46780027	9.634005671	0.001
rs192370125	KIRREL3	IN_AutDB	rs1995994	SRGAP3	IN_AutDB	202	0.207920792	4.002475248	77	0.051948052	0.249845393	8.753478818	10.04016371	0.0011
rs2945389	KSR1	NOTIN	rs2475838	HIVEP3	IN_AutDB	202	0.079207921	0.321000521	77	0.246753247	3.11525974	12.78328693	23.66581964	0.0004
rs13134636	LINC02511	NOTIN	rs884366	CCDC162P	NOTIN	202	0.292079208	3.748349835	77	0.077922078	0.266784063	12.64910439	10.77131641	0.0001
rs9660492	MIR181A1HG	NOTIN	rs593443	TENM4	NOTIN	202	0.252475248	3.888118812	77	0.064935065	0.257193787	12.92956545	7.642954791	0.0004
rs2374653	MTERF2	NOTIN	rs34416783	TRPM6	NOTIN	202	0.207920792	3.201980198	77	0.064935065	0.312306741	8.753478818	8.849393801	0.0038
rs11882611	NLRP12	NOTIN	rs11633653	OR4H6P	NOTIN	202	0.212871287	4.097772277	77	0.051948052	0.244035035	9.193295296	13.98073351	0.001
rs221422	NRXN3	IN_AutDB	rs10245943	TNRC18	NOTIN	202	0.212871287	3.278217822	77	0.064935065	0.305043793	9.193295296	10.13457859	0.0025
rs221422	NRXN3	IN_AutDB	rs3775375	FAM13A	NOTIN	202	0.212871287	3.278217822	77	0.064935065	0.305043793	9.193295296	10.69818548	0.0025
rs3791149	PTPRF	NOTIN	rs10801032	LINC01035	NOTIN	202	0.217821782	4.193069307	77	0.051948052	0.238488784	9.63927677	19.86085542	0.0006
rs3791149	PTPRF	NOTIN	rs1995994	SRGAP3	IN_AutDB	202	0.326732673	4.193069307	77	0.077922078	0.238488784	16.75366259	7.844348417	<0.0001
rs892034	SIRT2	NOTIN	rs1015674	PTPRS	NOTIN	202	0.202970297	3.125742574	77	0.064935065	0.319923978	8.319903419	8.786132896	0.0061
rs1995994	SRGAP3	IN_AutDB	rs4973647	LINC01880	NOTIN	202	0.207920792	8.004950495	77	0.025974026	0.124922696	12.56229436	11.3435666	<0.0001
rs34416783	TRPM6	NOTIN	rs520210	NEDD4L	NOTIN	202	0.202970297	3.125742574	77	0.064935065	0.319923978	8.319903419	7.615367101	0.0061
rs12352790	UNC13B	NOTIN	rs1266441	TMCO4	NOTIN	202	0.079207921	0.321000521	77	0.246753247	3.11525974	12.78328693	12.37952636	0.0004
rs12352790	UNC13B	NOTIN	rs2399867	CAMK1D	NOTIN	202	0.074257426	0.317656766	77	0.233766234	3.148051948	12.11955972	14.60297346	0.0006
rs1004407	WDFY4	NOTIN	rs1479868	CLSTN2	IN_AutDB	202	0.054455446	0.262066832	77	0.207792208	3.815820543	13.2989486	10.28978202	0.0004

rs11635835	WDR72	NOTIN	rs13190040	MCC	IN_AutDB	202	0.059405941	0.269073966	77	0.220779221	3.716450216	13.90872146	21.91350318	0.0002

Contrast Subgroup 4:

Subgroup1: Low ABC III Stereotypy Scale AND Late to Use Words
Subgroup2: High ABC III Stereotypy Scale AND Late to Use Words

SNP_ID1	Gene Symbol1	In_AutDB?	SNP_ID2	Gene Symbol2	In_AutDB?	Group1 Size	Support1	Growth1	Goup2 Size	Support2	Growth2	Chi Square between two subgroups	Chi Square compared to family members	P-Value
rs4338529	ANO5	NOTIN	rs4327349	CLSTN2	IN_AutDB	171	0.064327485	0.28411306	159	0.226415094	3.519725557	16.42017344	9.900457167	<0.0001
rs62391601	ATP10B	NOTIN	rs11114679	ACSS3	NOTIN	171	0.070175439	0.328173375	159	0.213836478	3.047169811	13.00229304	25.49589261	0.0002
rs57028861	CCZ1B	NOTIN	rs6949932	RPL12P10	NOTIN	171	0.064327485	0.292230576	159	0.220125786	3.421955403	15.39737681	12.03321745	<0.0001
rs10426289	ECH1	NOTIN	rs55992304	PLA2R1	NOTIN	171	0.058479532	0.281765019	159	0.20754717	3.549056604	14.8669837	7.552807883	<0.0001
rs10772706	GRIN2B	IN_AutDB	rs2374653	MTERF2	NOTIN	171	0.064327485	0.319627193	159	0.201257862	3.12864494	12.45036949	10.8328945	0.0003
rs10772706	GRIN2B	IN_AutDB	rs507812	ASB1	NOTIN	171	0.052631579	0.253588517	159	0.20754717	3.943396226	16.43407769	4.733149606	<0.0001
rs1557052	HDDC2	NOTIN	rs6784585	CLSTN2	IN_AutDB	171	0.064327485	0.319627193	159	0.201257862	3.12864494	12.45036949	13.8715031	0.0003
rs6565896	LINC00908	NOTIN	rs2275623	HDDC2	NOTIN	171	0.064327485	0.319627193	159	0.201257862	3.12864494	12.45036949	11.9378842	0.0003
rs6565896	LINC00908	NOTIN	rs513748	TPD52L1	NOTIN	171	0.070175439	0.318796992	159	0.220125786	3.136792453	13.96478018	22.27010199	0.0001
rs4841073	RPL10P19	NOTIN	rs570110	TPD52L1	NOTIN	171	0.070175439	0.286099865	159	0.245283019	3.495283019	18.01799101	16.42966141	<0.0001
rs4841073	RPL10P19	NOTIN	rs1557052	HDDC2	NOTIN	171	0.070175439	0.293628809	159	0.238993711	3.405660377	16.97536885	14.5284865	<0.0001
rs6949932	RPL12P10	NOTIN	rs7201610	PIEZO1	NOTIN	171	0.070175439	0.328173375	159	0.213836478	3.047169811	13.00229304	13.55840428	0.0002
rs892034	SIRT2	NOTIN	rs875591	PDE10A	NOTIN	171	0.064327485	0.30994152	159	0.20754717	3.226415094	13.41179054	7.164625055	0.0002
rs570110	TPD52L1	NOTIN	rs4327349	CLSTN2	IN_AutDB	171	0.064327485	0.30994152	159	0.20754717	3.226415094	13.41179054	14.77859356	0.0002
rs9677002	ZNF578	NOTIN	rs62391601	ATP10B	NOTIN	171	0.076023392	0.326695116	159	0.232704403	3.06095791	14.53785998	20.38891159	<0.0001

Contrast Subgroup 5:

Subgroup1: Mid Vineland II Daily Living AND High Height Z Score AND High ADIR C Total
Subgroup2: High Vineland II Daily Living AND High Height Z Score AND High ADIR C Total

SNP_ID1	Gene Symbol1	In_AutDB?	SNP_ID2	Gene Symbol2	In_AutDB?	Group1 Size	Support1	Growth1	Goup2 Size	Support2	Growth2	Chi Square between two subgroups	Chi Square compared to family members	P-Value
rs2843566	AGAP7P	NOTIN	rs79715516	IMMP2L	IN_AutDB	253	0.205533597	5.549407115	54	0.037037037	0.18019943	7.595959569	6.673135096	0.0015
rs2509151	ANO1	NOTIN	rs2799240	NOTCH2	NOTIN	253	0.051383399	0.231225296	54	0.222222222	4.324786325	15.16164447	28.84349634	0.0002
rs4338529	ANO5	NOTIN	rs2216455	PKP4	NOTIN	253	0.213438735	5.76284585	54	0.037037037	0.173525377	8.143769335	7.734142628	0.0015
rs4660463	KCNQ4	NOTIN	rs71476227	ZDHHC21	NOTIN	253	0.217391304	5.869565217	54	0.037037037	0.17037037	8.421881397	27.06870068	0.0009
rs4660463	KCNQ4	NOTIN	rs6698338	KCNH1	NOTIN	253	0.213438735	11.5256917	54	0.018518519	0.086762689	10.2142929	12.49526888	0.0002
rs10975959	KDM4C	IN_AutDB	rs3884489	PRAMEF25	NOTIN	253	0.04743083	0.232842257	54	0.203703704	4.294753086	13.51404058	12.89413814	0.0005

rs6760065	KIAA1211L	NOTIN	rs6510635	BTBD2	NOTIN	253	0.205533597	5.549407115	54	0.037037037	0.18019943	7.595959569	8.445612116	0.0015
rs6082465	LINC01727	NOTIN	rs4467127	MYHAS	NOTIN	253	0.043478261	0.213438735	54	0.203703704	4.685185185	14.85634927	21.58090213	0.0003
rs13024749	LY75	NOTIN	rs7487942	LINC00937	NOTIN	253	0.035573123	0.174631692	54	0.203703704	5.726337449	17.99541076	10.00148586	<0.0001
rs3760429	MYH1	NOTIN	rs6082465	LINC01727	NOTIN	253	0.043478261	0.213438735	54	0.203703704	4.685185185	14.85634927	22.31563684	0.0003
rs11651216	MYH2	NOTIN	rs6082465	LINC01727	NOTIN	253	0.04743083	0.232842257	54	0.203703704	4.294753086	13.51404058	22.03648222	0.0005
rs34019240	PPM1E	NOTIN	rs6818637	TET2	IN_AutDB	253	0.264822134	7.150197628	54	0.037037037	0.139856274	11.98085955	11.3568099	0.0001
rs28913018	TPO	IN_AutDB	rs6510635	BTBD2	NOTIN	253	0.221343874	5.976284585	54	0.037037037	0.167328042	8.70280082	6.966862069	0.0009

Contrast Subgroup 6:

Subgroup1: Mid CBCL6 Rule Breaking Score AND Low CBCL6 Activities Score AND High SRS-P Total Score
Subgroup2: High CBCL6 Rule Breaking Score AND Low CBCL6 Activities Score AND High SRS-P Total Score

SNP_ID1	Gene Symbol1	In_AutDB?	SNP_ID2	Gene Symbol2	In_AutDB?	Group1 Size	Support1	Growth1	Goup2 Size	Support2	Growth2	Chi Square between two subgroups	Chi Square compared to family members	P-Value
rs11080665	LDLRAD4	NOTIN	rs35448252	LINC00607	NOTIN	228	0.030701754	0.150950292	59	0.203389831	6.624697337	19.91221763	15.65250561	<0.0001
rs185483880	PRKRIP1	NOTIN	rs12969427	KCNG2	NOTIN	228	0.048245614	0.218960864	59	0.220338983	4.567026194	15.94585056	25.61811398	0.0001
rs7618862	CNTN4	IN_AutDB	rs9677002	ZNF578	NOTIN	228	0.214912281	4.226608187	59	0.050847458	0.236596333	7.437778112	13.14058342	0.0022
rs1377570	CNTN5	IN_AutDB	rs12712037	KIAA1211L	NOTIN	228	0.276315789	5.434210526	59	0.050847458	0.18401937	12.21671702	8.633257806	<0.0001
rs6797228	FHIT	IN_AutDB	rs9677002	ZNF578	NOTIN	228	0.206140351	6.081140351	59	0.033898305	0.164442842	8.646514627	7.299263686	0.0008
rs11657484	GGA3	NOTIN	rs10416031	POLR2E	NOTIN	228	0.043859649	0.215643275	59	0.203389831	4.637288136	14.6821069	17.79233852	0.0002
rs2727726	HSPB8	NOTIN	rs1960546	LINC00970	NOTIN	228	0.052631579	0.221804511	59	0.237288136	4.508474576	17.22994972	17.89717504	<0.0001
rs12316998	KDM5A	NOTIN	rs7183307	LINS1	NOTIN	228	0.048245614	0.237207602	59	0.203389831	4.215716487	13.27870725	26.30959012	0.0004
rs652092	MIR4300HG	NOTIN	rs13009166	EFHD1	NOTIN	228	0.048245614	0.237207602	59	0.203389831	4.215716487	13.27870725	9.35643478	0.0004
rs55882154	OXA1L	NOTIN	rs34416783	TRPM6	NOTIN	228	0.219298246	4.312865497	59	0.050847458	0.231864407	7.753598339	16.01011302	0.0022
rs55882154	OXA1L	NOTIN	rs9400305	AK9	NOTIN	228	0.214912281	4.226608187	59	0.050847458	0.236596333	7.437778112	22.09625369	0.0022
rs55882154	OXA1L	NOTIN	rs13103161	RN7SL89P	NOTIN	228	0.219298246	4.312865497	59	0.050847458	0.231864407	7.753598339	7.780164413	0.0022
rs55882154	OXA1L	NOTIN	rs7661349	TET2	IN_AutDB	228	0.23245614	4.571637427	59	0.050847458	0.218740006	8.72470403	7.711312556	0.0008
rs6797484	PDZRN3	NOTIN	rs12449479	MYOCD	NOTIN	228	0.048245614	0.218960864	59	0.220338983	4.567026194	15.94585056	29.21891923	0.0001
rs7661349	TET2	IN_AutDB	rs6053147	CDS2	NOTIN	228	0.23245614	4.571637427	59	0.050847458	0.218740006	8.72470403	15.3625757	0.0008
