# Supplementary material for: Exploratory Data Mining for Subgroup Cohort Discoveries and Prioritization
Source: IEEE J Biomed Health Inform. Author manuscript; Available in PMC 2022 Aug 1. (PMC9341221; doi:10.1109/JBHI.2019.2939149)
Supplement: Supplement 2 [file NIHMS1822002-supplement-Supplement_2.pdf]

Supplement 2

This document contains the search queries of six contrast subgroups which were used to search the articles in the PubMed. The numbers of PubMed articles listed in Table II were obtained from the search results.

Contrast Subgroup ID	Subgroup 1 Name	Subgroup 2 Name	Search Query in PubMed
1	Low SSC Full Scale IQ	High SSC Full Scale IQ	(autism OR asd)<br>AND<br>(IQ OR "intelligence quotient")
2	Normal to Speak Sentences	Late to Speak Sentences	(autism OR asd)<br>AND<br>("language development" OR communication deficit* OR conversation OR expressive OR linguistic OR speech)
3	Mid RBS-R Overall Score<br>**AND**<br>Low CBCL6 Social Score	Low RBS-R Overall Score<br>**AND**<br>Low CBCL6 Social Score	(autism OR asd)<br>AND<br>(repetitive behavior* OR restricted behavior* OR stereotyped behavior* OR self-injurious behavior*OR compulsive behavior* OR routine behavior* OR sameness behavior* OR restricted behavior* OR repetitive behaviour* OR restricted behaviour* OR stereotyped behaviour* OR self-injurious behaviour*OR compulsive behaviour* OR routine behaviour* OR sameness behaviour* OR restricted behaviour*)<br>AND<br>(impaired social function* OR social impairment* OR social interaction* OR social communication deficit* OR communicative deficits* OR socialisation* OR socialization* OR social deficit* OR friendship* OR CBCL6 social score*)
4	Low ABC III Stereotypy Scale<br>**AND**<br>Late to Use Words	High ABC III Stereotypy Scale<br>**AND**<br>Late to Use Words	(autism OR asd)<br>AND<br>(stereotypical behavior* OR perseveration* OR self-stimulatory behavior* OR stimming behavior*OR ritualistic behavior* OR repetitive behavior* OR restricted behavior* OR stereotyped behavior* OR self-injurious behavior* OR compulsive behavior* OR routine behavior* OR sameness behavior*

			OR stereotypical behaviour* OR self-stimulatory behaviour* OR stimming behaviour* OR ritualistic behaviour* OR repetitive behaviour* OR restricted behaviour* OR stereotyped behaviour* OR self-injurious behaviour* OR compulsive behaviour* OR routine behaviour* OR sameness behaviour*)<br>AND<br>("word delay" OR "delayed speech" OR "language development" OR communication deficit* OR conversation OR expressive OR linguistic OR speech OR "delayed echolalia")
5	Mid Vineland II Daily Living<br>**AND**<br>High Height Z Score<br>**AND**<br>High ADIR C Total	High Vineland II Daily Living<br>**AND**<br>High Height Z Score<br>**AND**<br>High ADIR C Total	(autism OR asd)<br>AND<br>("daily living" OR adaptive function impairment* OR adaptive skill*)<br>AND<br>(Height)<br>AND<br>(stereotypical behavior* OR perseveration* OR self-stimulatory behavior* OR stimming behavior*OR ritualistic behavior* OR repetitive behavior* OR restricted behavior* OR stereotyped behavior* OR self-injurious behavior* OR compulsive behavior* OR routine behavior* OR sameness behavior*<br>OR restricted behavior* OR circumscribed interest* OR encompassing preoccupation*OR stereotypical behaviour* OR self-stimulatory behaviour* OR stimming behaviour*OR ritualistic behaviour* OR repetitive behaviour* OR restricted behaviour* OR stereotyped behaviour* OR self-injurious behaviour* OR compulsive behaviour* OR routine behaviour* OR sameness behaviour*OR restricted behaviour*)
6	Mid CBCL6 Rule Breaking Score<br>**AND**<br>Low CBCL6 Activities Score<br>**AND**<br>High SRS-P Total Score	High CBCL6 Rule Breaking Score<br>**AND**<br>Low CBCL6 Activities Score<br>**AND**<br>High SRS-P Total Score	(autism OR asd)<br>AND<br>("rule breaking" OR "alcohol use" OR "tobacco" OR use drug*)<br>AND<br>("exploratory activity" OR "activity participation" OR "sports" OR "CBCL6 Activities")<br>AND<br>("social responsiveness" OR "social awareness" OR social impairment* OR "social information processing" OR reciprocal social communication* OR "social anxiety" OR "social avoidance")
