# Supplementary material for: Exploratory Data Mining for Subgroup Cohort Discoveries and Prioritization
Source: IEEE J Biomed Health Inform. Author manuscript; Available in PMC 2022 Aug 1. (PMC9341221; doi:10.1109/JBHI.2019.2939149)
Supplement: Supplement 3 [file NIHMS1822002-supplement-Supplement_3.pdf]

Supplement 3

This table lists top 30 Autism subgroups with its J-value. The J-values range from 2.68 to 17.56 for the top 10 single-population variable subgroups. For double- and triple-population variable subgroups, the J-values increase due to the facts that when subgroups are tailored to more specific populations, the homogeneities of patterns with each subgroup get higher. To present the results to the users, it is recommended to rank/prioritize subgroups separately based on the number of population variables.

ID	Subgroup 1 Name	Cohort 1 Size	Subgroup 2 Name	Cohort 2 Size	J-value
**Single-Population-Variable Subgroup Pairs:**					
1	Normal to Speak Sentences	346	Late to Speak Sentences	304	17.56
2	Low SSC Full Scale IQ	459	High SSC Full Scale IQ	373	14.39
3	Low SRS-P Total Score	120	High SRS-P Total Score	1781	13.28
4	Low SSC Overall verbal IQ	507	High SSC Overall verbal IQ	365	12.02
5	Low CBCL6 Activities Score	349	High CBCL6 Activities Score	270	7.54
6	Low CBCL6 Social Score	296	High CBCL6 Social Score	294	5.94
7	Low ADOS CSS RRB	294	High ADOS CSS RRB	444	2.89
8	Low Height Z Score	381	High Height Z Score	371	2.86
9	Low ABC III Stereotypy Scale	387	High ABC III Stereotypy Scale	403	2.78
10	Low Vineland II Daily Living	426	High Vineland II Daily Living	412	2.68
**Double-Population-Variable Subgroup Pairs:**					
1	Low ABC III Stereotypy Scale **AND** Late to Use Words	171	High ABC III Stereotypy Scale **AND** Late to Use Words	159	16.32
2	Mid RBS-R Overall Score **AND** Low CBCL6 Social Score	202	Low RBS-R Overall Score **AND** Low CBCL6 Social Score	77	15.39
3	Mid ADOS CSS RRB **AND** Low CBCL6 Activities Score	223	High ADOS CSS RRB **AND** Low CBCL6 Activities Score	74	15.37
4	Mid SSC Full Scale IQ **AND** High CBCL6 Rule Breaking Score	238	High SSC Full Scale IQ **AND** High CBCL6 Rule Breaking Score	59	15.37
5	Mid Vineland II Daily Living **AND** Late to Speak Sentences	211	Low Vineland II Daily Living **AND** Late to Speak Sentences	81	15.28
6	Normal to Speak Sentences **AND** Low ABC III Stereotypy Scale	224	Early to Speak Sentences **AND** Low ABC III Stereotypy Scale	63	15.18
7	Mid ABC III Stereotypy Scale **AND** Late to Speak Sentences	217	High ABC III Stereotypy Scale **AND** Late to Speak Sentences	57	14.95
8	Mid SSC Overall verbal IQ **AND**	203	High SSC Overall verbal IQ **AND**	71	14.95

	Low ADOS CSS RRB		Low ADOS CSS RRB		
9	Mid ABC III Stereotypy Scale **AND**<br>Low CBCL6 Social Score	200	Low ABC III Stereotypy Scale **AND**<br>Low CBCL6 Social Score	73	14.93
10	Mid SRS-P Total Score **AND**<br>Late to walked alone	67	High SRS-P Total Score **AND**<br>Late to walked alone	205	14.91
**Triple-Population-Variable Subgroup Pairs:**					
1	Mid Vineland II Daily Living **AND**<br>High Height Z Score **AND**<br>High ADIR C Total	253	High Vineland II Daily Living **AND**<br>High Height Z Score **AND**<br>High ADIR C Total	54	19.27
2	Mid CBCL6 Rule Breaking Score **AND**<br>Low CBCL6 Activities Score **AND**<br>High SRS-P Total Score	228	High CBCL6 Rule Breaking Score **AND**<br>Low CBCL6 Activities Score **AND**<br>High SRS-P Total Score	59	18.75
3	Mid SSC Full Scale IQ **AND**<br>High CBCL6 Rule Breaking Score **AND**<br>High ADIR C Total	227	High SSC Full Scale IQ **AND**<br>High CBCL6 Rule Breaking Score **AND**<br>High ADIR C Total	52	18.54
4	Mid CBCL6 Activities Score **AND**<br>High SSC Overall verbal IQ **AND**<br>High ADIR C Total	213	High CBCL6 Activities Score **AND**<br>High SSC Overall verbal IQ **AND**<br>High ADIR C Total	51	18.46
5	Mid RBS-R Overall Score **AND**<br>Low Height Z Score **AND**<br>High SRS-P Total Score	207	High RBS-R Overall Score **AND**<br>Low Height Z Score **AND**<br>High SRS-P Total Score	55	18.41
6	Mid RBS-R Overall Score **AND**<br>Early to walked alone **AND**<br>High SSC Overall verbal IQ	214	Low RBS-R Overall Score **AND**<br>Early to walked alone **AND**<br>High SSC Overall verbal IQ	59	18.38
7	Mid ABC III Stereotypy Scale **AND**<br>Late to Speak Sentences **AND**<br>High ADIR C Total	213	High ABC III Stereotypy Scale **AND**<br>Late to Speak Sentences **AND**<br>High ADIR C Total	57	18.29
8	Mid ADOS CSS RRB **AND**<br>Early to walked alone **AND**<br>Early to Speak Sentences	219	Low ADOS CSS RRB **AND**<br>Early to walked alone **AND**<br>Early to Speak Sentences	50	18.27
9	Mid CBCL6 Social Score **AND**<br>Low CBCL6 Activities Score **AND**<br>Mid CBCL6 Rule Breaking Score	214	Low CBCL6 Social Score **AND**<br>Low CBCL6 Activities Score **AND**<br>Mid CBCL6 Rule Breaking Score	52	18.18
10	Mid ADOS CSS RRB **AND**<br>Low CBCL6 Activities Score **AND**<br>High SRS-P Total Score	188	High ADOS CSS RRB **AND**<br>Low CBCL6 Activities Score **AND**<br>High SRS-P Total Score	59	17.96
