# Supplementary material for: Exploratory Data Mining for Subgroup Cohort Discoveries and Prioritization
Source: IEEE J Biomed Health Inform. Author manuscript; Available in PMC 2022 Aug 1. (PMC9341221; doi:10.1109/JBHI.2019.2939149)
Supplement: Supplement 4 [file NIHMS1822002-supplement-Supplement_4.pdf]

Supplement 4

This supplement document contains comparisons of the hierarchical clustering [46], network analysis [47], and our Guided Cascading Shotgun Method on both synthesized data set and Autism data set.

A. Synthetic Data – Comparison with Unsupervised Machine Learning Methods

To conduct a fair comparison of existing unsupervised machine learning methods for cohort discovery as discussed in Section II, we chose the synthesized data set with five population variables ($|P| = 5$) and 10 pairs of population subgroups ($N = 10$) used in Section VI.A. To ensure clear visualization of the results from the two unsupervised learning methods, we down sampled the population size to 4,000 and kept the original distribution of subgroups. The evaluations were based on the success of identifying population subgroups, e.g. "BMI >25, Gender=Male", Blood Pressure is high (>160), using a coverage measurement which is the ratio of discovered population subgroups and those assigned in the synthesized data set. A 100% coverage means that the algorithm is able to find clusters representing all 10 pairs of population subgroups (20 population subgroups).

Hierarchical Clustering:

We applied Gower distance to calculate the distance matrix on categorical measurement variables and use hclust() function in R to cluster the data. The dendrogram of all subgroups is shown in Fig. S1. We set the number of clusters to 20 as we predefined in the synthesized data (10 pairs of population subgroups). We then found the top population representation from each cluster and matched it with the 10-pair synthesized subpopulations. 7 out of 20 (Coverage: 35%) population subgroups were discovered by the hierarchical clustering method.

Network Analysis:

In the network analysis, we also calculated the Gower distance matrix of data points and kept the edges if the dissimilarity of two points is lower than 0.5 (distance threshold). We used 'igraph' package in R and implemented the fast-greedy modularity optimization algorithm to find the clusters in the graph. The network with 20 subgroups is shown in Fig. S2. We also found the top population representation from each cluster and matched it with the 10-pair synthesized subpopulations. 9 out of 20 (Coverage: 45%) population subgroups were discovered by the network analysis method.

Guided Cascading Shotgun Method:

To compare with hierarchical clustering and network analysis, we applied the Guided Cascading Shotgun approach to the synthesized data set. Our method discovered 14 population subgroups within top 20 subgroups (Coverage: 70%). Table S.I lists the comparison of population coverage with the two unsupervised clustering methods. It is noteworthy to mention that the results from the unsupervised learning methods are clusters that are individually separable from each other but do not provide contrast patterns that might be valuable for biomedical applications between cohorts.

TABLE S.I

COMPARISON OF POPULATION COVERAGE

Methods	Guided Cascading Shotgun	Hierarchical Clustering	Network Analysis
Coverage	70%	35%	45%

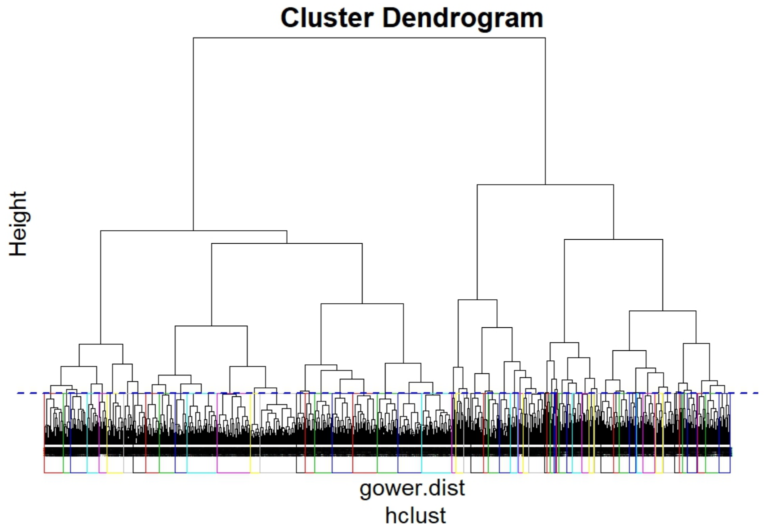

Fig. S.1. A hierarchical clustering dendrogram of synthesized data with 20 population subgroups.

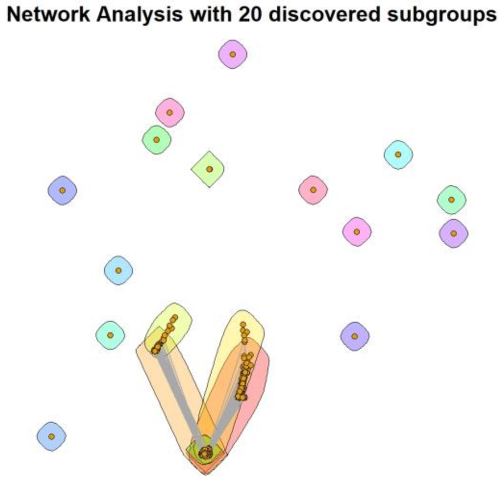

Fig. S.2. A graph of network analysis with 20 population subgroups.

B. Autism Data Set – Comparison with Unsupervised Machine Learning Methods

We applied the hierarchical clustering and network analysis on the Autism dataset using the Manhattan distance. For the hierarchical clustering method, we specified 3 to 10 clusters. We mined frequent patterns of phenotype combinations as subgroups within each cluster and then did the Fisher's exact test to assess statistically significant subgroups of each cluster compared to the rest of the population. As shown in Table S.II, the hierarchical clustering method resulted in only six significant subgroups. Two of these subgroups, "Low SSC Overall verbal IQ" and "Low SSC Full Scale IQ", were also found by our Exploratory Data Mining method with 2nd and 4th

ranks in the Single-Population-Variable Subgroup Pairs reported in Supplement 3. Of the other four subgroups, two of them, "Mid CBCL6 Activities Score" and "Early to Use Word", do not have significant genes that frequently appear in the subgroup but not the rest of the population. However, the hierarchical clustering method found the subgroup "Late to Use Word" and "Low SSC Full Scale IQ **AND** Low SSC Overall verbal IQ" which were not included in our ranked results (top 142). There are few reasons for such discrepancy. For "Late to Use Word" subgroup, the sample size and the number of significant genes did not contribute enough to the J-value in our exploratory mining process. Therefore, the subgroup was not included in the ranked results.

For network analysis, we also picked 3-10 clusters and mined frequent phenotype combinations as subgroups within the cluster. There are only two major subgroup types. The subgroup "Late to Use Word" has 59 patients with 17 significant genes inside the subgroup. It is noteworthy to mention that there are 306 patients with the same phenotype residing in other clusters. The subgroup "Mid Height Z Score" was also found by the network analysis method. However, the subgroup did not have significant genes compared to the rest of the population.

The subgroups identified by the hierarchical clustering and network analysis are highlighted in Table S.III. This experiment shows that the phenotype/population frequent pattern extraction from both hierarchical clustering and network analysis methods identify a very small portion of the subgroup cohorts identified by our exploratory mining method.

TABLE S.II

Methods	Subgroup Name	Are there any significant genes compared to the rest of data?	Is it found by our Exploratory Data Mining? (If yes, rank?)
Hierarchical Clustering	Low SSC Overall verbal IQ	Yes	Yes (2nd ranked of Single-Population-Variable Subgroup Pairs)
	Low SSC Full Scale IQ	Yes	Yes (4th ranked of Single-Population-Variable Subgroup Pairs)
	Mid CBCL6 Activities Score	No	No
	Early to Use Word	No	No
	Late to Use Word	Yes	No
	Low SSC Full Scale IQ **AND** Low SSC Overall verbal IQ	Yes	No
Network Analysis	Late to Use Word	No	No
	Mid Height Z Score	No	No

TABLE S.III

No. of Clusters	Subgroup Name	Subgroup Size (Percentage in a Sub-cluster)	P-value of Subgroup	No. of Significant Genes compared to the rest of data
Hierarchical Clustering				

3	Low SSC Overall verbal IQ	16 (43.24%)	0.0011	122
4	Low SSC Overall verbal IQ	16 (43.24%)	0.0011	122
5	Mid CBCL6 Activities Score	1249 (52.68%)	0.0002	0
5	Early to Use Word	1008 (42.51%)	0.001	0
5	Late to Use Word	59 (62.11%)	0.0001	17
5	Low SSC Overall verbal IQ	16 (43.24%)	0.0011	122
6	Mid CBCL6 Activities Score	1249 (52.68%)	0.0002	0
6	Early to Use Word	1008 (42.51%)	0.001	0
6	Late to Use Word	59 (62.11%)	0.0001	17
6	Low SSC Overall verbal IQ	16 (43.24%)	0.0011	122
7	Mid CBCL6 Activities Score	1249 (52.68%)	0.0002	0
7	Early to Use Word	1008 (42.51%)	0.001	0
7	Late to Use Word	59 (62.11%)	0.0001	17
7	Low SSC Overall verbal IQ	16 (43.24%)	0.0011	122
7	Low SSC Full Scale IQ	15 (45.45%)	0.0002	14
7	Low SSC Overall verbal IQ	14 (42.42%)	0.0028	21
8	Mid CBCL6 Activities Score	1249 (52.68%)	0.0002	0
8	Early to Use Word	1008 (42.51%)	0.001	0
8	Late to Use Word	59 (62.11%)	0.0001	17
8	Low SSC Overall verbal IQ	13 (61.90%)	<0.0001	114
8	Low SSC Full Scale IQ	11(52.38%)	0.0003	124
8	Low SSC Full Scale IQ **AND** Low SSC Overall verbal IQ	11(52.38%)	0.0012	124
8	Low SSC Full Scale IQ	15 (45.45%)	0.0002	14
8	Low SSC Overall verbal IQ	14 (42.42%)	0.0028	21
9	Mid CBCL6 Activities Score	1249 (52.68%)	0.0002	0
9	Early to Use Word	1008 (42.51%)	0.001	0
9	Late to Use Word	59 (62.11%)	0.0001	17
9	Low SSC Overall verbal IQ	13 (61.90%)	<0.0001	114
9	Low SSC Full Scale IQ	11(52.38%)	0.0003	124
9	Low SSC Full Scale IQ **AND** Low SSC Overall verbal IQ	11(52.38%)	0.0012	124
9	Low SSC Full Scale IQ	15 (45.45%)	0.0002	14
9	Low SSC Overall verbal IQ	14 (42.42%)	0.0028	21
10	Mid CBCL6 Activities Score	1249 (52.68%)	0.0002	0
10	Early to Use Word	1008 (42.51%)	0.001	0

10	Late to Use Word	59 (62.11%)	0.0001	17
10	Low SSC Overall verbal IQ	12 (60.00%)	<0.0001	130
10	Low SSC Full Scale IQ	10 (50.00%)	0.0009	134
10	Low SSC Full Scale IQ **AND** Low SSC Overall verbal IQ	10 (50.00%)	0.0032	134
10	Low SSC Full Scale IQ	15 (45.45%)	0.0002	14
10	Low SSC Overall verbal IQ	14 (42.42%)	0.0028	21
Network Analysis				
3	Late to Use Word	544 (46.10%)	0.0005	0
4	Late to Use Word	544 (46.10%)	0.0005	0
5	Late to Use Word	233 (48.85%)	0.0017	1
6	Late to Use Word	233 (48.85%)	0.0017	1
7	Late to Use Word	233 (48.85%)	0.0017	1
7	Mid Height Z Score	252 (75.22%)	0.0079	0
8	Late to Use Word	233 (48.85%)	0.0017	1
8	Mid Height Z Score	252 (75.22%)	0.0079	0
9	Mid Height Z Score	252 (75.22%)	0.0079	0
9	Late to Use Word	99 (51.83%)	0.0062	17
10	Mid Height Z Score	252 (75.22%)	0.0079	0
10	Late to Use Word	99 (51.83%)	0.0062	17
